# Unravelling the Genome-Wide Contributions of Specific 2-Alkyl-4-Quinolones and PqsE to Quorum Sensing in *Pseudomonas aeruginosa*

**DOI:** 10.1371/journal.ppat.1006029

**Published:** 2016-11-16

**Authors:** Giordano Rampioni, Marilena Falcone, Stephan Heeb, Emanuela Frangipani, Matthew P. Fletcher, Jean-Frédéric Dubern, Paolo Visca, Livia Leoni, Miguel Cámara, Paul Williams

**Affiliations:** 1 Department of Science, University Roma Tre, Rome, Italy; 2 School of Life Sciences, Centre for Biomolecular Sciences, University of Nottingham, Nottingham, United Kingdom; East Carolina University School of Medicine, UNITED STATES

## Abstract

The *pqs* quorum sensing (QS) system is crucial for *Pseudomonas aeruginosa* virulence both *in vitro* and in animal models of infection and is considered an ideal target for the development of anti-virulence agents. However, the precise role played by each individual component of this complex QS circuit in the control of virulence remains to be elucidated. Key components of the *pqs* QS system are 2-heptyl-4-hydroxyquinoline (HHQ), 2-heptyl-3-hydroxy-4-quinolone (PQS), 2-heptyl-4-hydroxyquinoline *N*-oxide (HQNO), the transcriptional regulator PqsR and the PQS-effector element PqsE. To define the individual contribution of each of these components to QS-mediated regulation, transcriptomic analyses were performed and validated on engineered *P*. *aeruginosa* strains in which the biosynthesis of 2-alkyl-4-quinolones (AQs) and expression of *pqsE* and *pqsR* have been uncoupled, facilitating the identification of the genes controlled by individual *pqs* system components. The results obtained demonstrate that *i)* the PQS biosynthetic precursor HHQ triggers a PqsR-dependent positive feedback loop that leads to the increased expression of only the *pqsABCDE* operon, *ii)* PqsE is involved in the regulation of diverse genes coding for key virulence determinants and biofilm development, *iii)* PQS promotes AQ biosynthesis, the expression of genes involved in the iron-starvation response and virulence factor production *via* PqsR-dependent and PqsR-independent pathways, and *iv)* HQNO does not influence transcription and hence does not function as a QS signal molecule. Overall this work has facilitated identification of the specific regulons controlled by individual *pqs* system components and uncovered the ability of PQS to contribute to gene regulation independent of both its ability to activate PqsR and to induce the iron-starvation response.

## Author Summary

Many bacterial pathogens control virulence gene expression and the development of antibiotic-resistant biofilms *via* intercellular communication through ‘quorum sensing’ (QS). QS systems depend on the synthesis, secretion and perception of diffusible signalling molecules that enable bacteria to synchronize their behaviour at the population level and are considered ideal targets for the development of anti-virulence drugs. *Pseudomonas aeruginosa* employs several overlapping QS circuits including the *pqs* system to control the expression of virulence determinants. The *pqs* QS system relies on multiple 2-alkyl-4-quinolones (AQs), including the *Pseudomonas* Quinolone Signal (PQS), as signal molecules. However, the individual contributions of key AQs and the effector proteins PqsR and PqsE within the auto-regulated *pqs* system have not been elucidated because of their inter-dependence. By constructing *P*. *aeruginosa* strains with multiple mutations in the *pqs* system and determining their transcriptomes in the presence or absence of PqsR, PqsE or exogenously supplied AQs, we define the distinct regulons involved and characterize a novel PQS signalling pathway independent of PqsR and the iron-starvation response.

## Introduction

*Pseudomonas aeruginosa* is a multi-antibiotic resistant pathogen commonly responsible for hospital-acquired infections and is the main cause of morbidity and mortality in cystic fibrosis [1]. The pathogenicity of *P*. *aeruginosa* is multifactorial and host specific relying on the coordinated production of multiple virulence factors and the formation of antibiotic tolerant biofilms [2,3]. These are controlled by a quorum sensing (QS) intercellular communication network that integrates information on population structure/dynamics and the metabolic status of the cell with environmental cues [4–7]. Since *P*. *aeruginosa* QS mutants display attenuated pathogenicity, QS is a promising target for anti-virulence agents [8].

In *P*. *aeruginosa* QS involves three major inter-linked QS signalling pathways, namely the *las* and *rhl* systems that employ *N*-acylhomoserine lactones and the *pqs* QS system that uses 2-alkyl-4-quinolones (AQs) as QS signal molecules [5]. Data from expression studies and virulence factor profiling obtained by comparing wild type with different *pqs* mutants have revealed the extent of the *pqs* regulon and its relationship with the *las* and *rhl* regulons. For example, AQs are required for full transcription of genes coding for exoenzymes, exotoxins, lectins, secondary metabolites (*e*.*g*., pyocyanin, hydrogen cyanide, rhamnolipids, pyochelin and pyoverdine) and biofilm development (reviewed in [9]). *P*. *aeruginosa* mutants defective in AQ biosynthesis or sensing are severely attenuated in plant and animal infection models [3,10,11]. Furthermore, AQs are detectable in sputum, blood and urine of individuals with cystic fibrosis and their presence correlates with clinical status [12].

**Fig 1:**
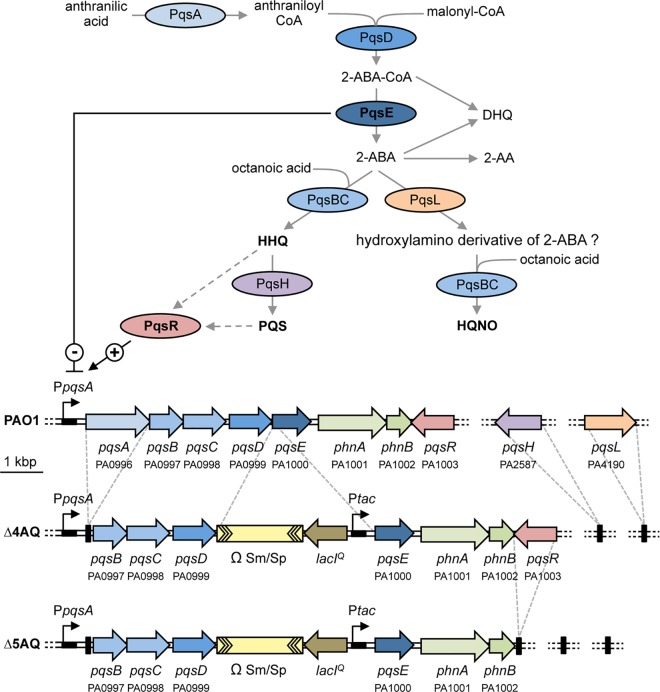
The AQ biosynthetic pathway and *pqs* genes. Schematic representation of the AQ biosynthetic pathway and the *pqs* and *phn* genes in *P*. *aeruginosa* PAO1 and the isogenic ∆4AQ and ∆5AQ mutants. Main elements of the *pqs* QS system (HHQ, PQS, HQNO, PqsE, and PqsR) are in bold face. The PA number is indicated below the genes according to the *Pseudomonas* Genome Database [13]. Solid grey arrows represent biosynthesis; dashed grey arrows represent information flow; solid black arrow indicates activation (+); black T-line indicates negative regulation (-).

The *pqs* system incorporates at least four transcriptional units, with *pqsABCDE* (PA0996-PA1000) and *pqsR* (PA1003) clustering at the same genetic locus, while *pqsH* (PA2587) and *pqsL* (PA4190) are distally located [13]. Our understanding of the molecular mechanisms governing *pqs-*dependent QS is however limited, largely because of the inter-dependent, auto-regulatory, multi-component nature of the system (**Fig 1**) [9].

*P*. *aeruginosa* produces >50 different AQs [14] of which 2-heptyl-3-hydroxy-4-quinolone (also known as the *Pseudomonas* Quinolone Signal, PQS) and its immediate precursor 2-heptyl-4-hydroxyquinoline (HHQ) are most closely associated with QS signalling. Most of the genes required for AQ biosynthesis are located in the *pqsABCDE* operon (**Fig 1**). PqsA converts anthranilic acid to anthraniloyl-CoA that is condensed with malonyl-CoA to form 2-aminobenzoylacetyl-CoA (2-ABA-CoA) in a reaction catalysed by PqsD [15,16]. The thioesterase activity of PqsE converts 2-ABA-CoA into 2-aminobenzoylacetate (2-ABA) [17]; HHQ is formed through the condensation of octanoyl-coenzyme A and 2-ABA *via* the PqsBC heterodimer [18,19].

Additional enzymes are required for the biosynthesis of PQS and 2-heptyl-4-hydroxyquinoline *N*-oxide (HQNO) (**Fig 1**). Under aerobic conditions, HHQ is oxidized to PQS *via* the action of the monooxygenase PqsH [20]. A second monooxygenase, PqsL, is required together with the *pqsABCD* gene products for the synthesis of HQNO and related *N*-oxides [21].

The *pqs* system is subject to positive autoregulation, since the LysR-type transcriptional regulator PqsR (MvfR), binds to the promoter region of *pqsABCDE* (P*pqsA*) and triggers transcription once activated by HHQ or PQS (**Fig 1**) [22–24]. Therefore, by analogy with other QS systems, HHQ and PQS act as autoinducers by generating a positive feedback loop that accelerates their biosynthesis. Although both HHQ and PQS can function as QS signal molecules, it is not clear whether this regulatory effect is only exerted *via* PqsR, or also *via* PqsR-independent pathways. Although both HHQ and PQS activate PqsR, PQS has additional properties. For example, PQS promotes the formation of membrane vesicles (MVs) in which PQS is both bioactive and bioavailable [25], although it is not essential for MV formation [26]. The 3-hydroxy substituent also confers on PQS the ability to chelate ferric iron (Fe^3+^) [27]. Consequently, exogenous PQS triggers an iron-starvation response in *P*. *aeruginosa*, promoting the production of the siderophores, pyoverdine and pyochelin [27,28]. However, PQS cannot be considered as a siderophore *sensu stricto*, since it does not stimulate growth of a siderophore-defective *P*. *aeruginosa* mutant in iron-deficient growth conditions [27]. PQS appears instead to act as an iron trap associated with the outer membrane. In this context, the iron-chelating property of PQS may confer a survival advantage to *P*. *aeruginosa* in mixed bacterial populations by limiting the availability of iron to co-inhabitant species [29].

HQNO also contributes to the environmental competitiveness of *P*. *aeruginosa*, since it is a potent inhibitor of the cytochrome *bc*_*1*_ complex [30]. At present, the role played by HQNO in *P*. *aeruginosa* physiology and the mechanism by which HQNO self-poisoning is avoided, have not been determined.

Mutations in the *pqsA*, *pqsB*, *pqsC* or *pqsD* biosynthetic genes or in the regulatory gene *pqsR*, all abolish AQ production, while *P*. *aeruginosa pqsH* and *pqsL* mutants accumulate either HHQ and HQNO or HHQ and PQS, respectively [9,14,31]. Notably, while PqsE converts 2-ABA-CoA to 2-ABA, a mutation in *pqsE* does not affect AQ biosynthesis [11]. This is probably because the PqsE thioesterase functionality can be provided by alternative thioesterases [17]. PqsE over-expression however completely abrogates P*pqsA* activity (**Fig 1**), and consequently AQ biosynthesis [11].

Although PqsE is dispensable for AQ biosynthesis, it is required for production of key virulence factors, such as pyocyanin, elastase, rhamnolipids, hydrogen cyanide, LecA lectin, and for biofilm maturation [11]. The activity of PqsE is also dependent on the *N*-butanoyl-homoserine lactone (C_4_-HSL) receptor RhlR, which acts downstream but in synergy with PqsE [32]. Therefore, it is likely that PqsE has, as yet unidentified, functions in addition to its thioesterase activity [17]. Transcriptomic analyses have revealed that the expression of multiple genes requires *pqsE*, and that full virulence in plant and animal infection models is strongly dependent on this enzyme [6,11]. Although the crystal structure of PqsE has been solved and key active site residues identified, the mechanism by which it controls *P*. *aeruginosa* virulence gene expression is not understood [17,33].

Since the HHQ- and PQS-dependent activation of *pqsABCDE* transcription results in increased levels of both AQs and PqsE, it is possible that functional effects previously considered to be HHQ- and/or PQS-dependent are mediated *via* PqsE. Alternatively, since PqsE over-expression abrogates AQ biosynthesis, some phenotypes altered as a consequence of increased *pqsE* expression may, at least in part, be under the control of HHQ and/or PQS. PqsE controls the expression of some virulence genes independent of contribution to AQ biosynthesis. The major reductions in LecA and pyocyanin production in an AQ-negative *pqsA* mutant for example could be restored fully by expressing *pqsE* from an inducible *tac* promoter [11]. Thus, the autoregulatory effect exerted by PqsE on its own transcription plays a homeostatic role in limiting AQ accumulation, thus impeding a clear understanding of the physiological role(s) played by PqsE.

**Fig 1** shows how the *pqs* system components are interlinked. HHQ and PQS both induce transcription of the *pqsABCDE* operon *via* PqsR, increasing AQ biosynthesis and *pqsE* expression. The latter in turn, exerts a repressive role on both AQ production and its own expression. This complexity has obscured comprehension of the physiological roles played by specific AQs and PqsE. Characterization of the regulons controlled by individual components of the *pqs* system has not yet been reported. For example, the genes controlled *via* the *pqs* system have been investigated by comparing the transcriptional profiles of *P*. *aeruginosa* PA14 wild type and its *pqsH* isogenic mutant [23] by evaluating the effect of exogenous PQS on the *P*. *aeruginosa* PAO1 transcriptome [28] or by comparing the wild type PAO1 with *pqsA* or *pqsE* mutants [11]. In each case, numerous genes including those involved in virulence factor production, iron homeostasis and denitrification, appeared to be PQS-controlled. However, since, in the strains used, altered PQS levels led to dysregulation of HHQ and HQNO synthesis and *pqsE* expression, it is not possible to discriminate between the role(s) played by PQS from that of the other components of the *pqs* system. Similarly, it is not possible to determine whether PqsE-controlled genes in strains overexpressing this protein are controlled by PqsE itself or by the lack of AQs resulting from *pqsE* overexpression [11].

To circumvent these limitations, a *P*. *aeruginosa* PAO1 mutant unable to synthesize AQs or convert exogenously supplied AQs was constructed. In this strain, termed ∆4AQ, *pqsE* expression is chemically inducible and uncoupled from the activity of the P*pqsA* promoter, thus exogenous AQ provision does not alter PqsE levels. Transcriptomic analyses were performed on the ∆4AQ strain grown in the absence or in the presence of either HHQ, PQS or HQNO, or the exogenous inducer of PqsE expression (IPTG), thus enabling identification of the specific genes controlled by each *pqs* system component. Transcriptomic analyses were also performed on strains with *pqsR*-proficient or *pqsR*-deficient (∆5AQ) genetic backgrounds to elucidate the physiological role(s) played by the transcriptional regulator PqsR.

## Results and Discussion

### Characterization of HHQ, PQS, HQNO and PqsE regulons

To identify the regulons controlled individually by HHQ, PQS, HQNO and PqsE, a quadruple mutant of *P*. *aeruginosa* PAO1, named ∆4AQ, was constructed. As depicted in **Fig 1**, this carries in frame deletions of *pqsA*, *pqsH* and *pqsL* genes, and incorporates an isopropyl β-d-l-thiogalactopyranoside (IPTG)-inducible *pqsE* gene. Preliminary experiments were performed to validate the ∆4AQ strain. *P*. *aeruginosa* PAO1 wild type was grown in LB, while the isogenic ∆4AQ mutant was grown in LB or in LB supplemented with either HHQ, PQS, or HQNO (40 μM), or with IPTG (1 mM). All strains were grown to late exponential phase where the *pqs* system is maximally expressed [11]. Cell-free spent media and bacterial cells were respectively collected for determination of AQ levels by LC-MS/MS, and for quantification of *pqsE* mRNA levels by Real Time PCR. HHQ, PQS and HQNO were only recovered from the ∆4AQ cultures if exogenously added, and were not converted into other AQs (**S1A Fig**). Moreover, the *P*. *aeruginosa* ∆4AQ strain grown in the absence of IPTG showed only basal levels of *pqsE* RNA (∆4AQ to wild type ratio ~ 0.2), irrespective of the presence or absence of AQs while IPTG addition increased *pqsE* RNA levels by ~15-fold relative to the parental strain (**S1B Fig**) [11]. Growth of the ∆4AQ strain was not affected by exogenous provision of any AQ or IPTG (**S1C Fig**).

The transcriptional profiles of the ∆4AQ strain grown with 40 μM of HHQ, PQS or HQNO or with IPTG were compared by means of high-density oligonucleotide microarrays, using Affymetrix GeneChip for *P*. *aeruginosa* PAO1. This method was chosen to provide a reliable comparison with previously published data [6,10,11,23,28].

**Fig 2:**
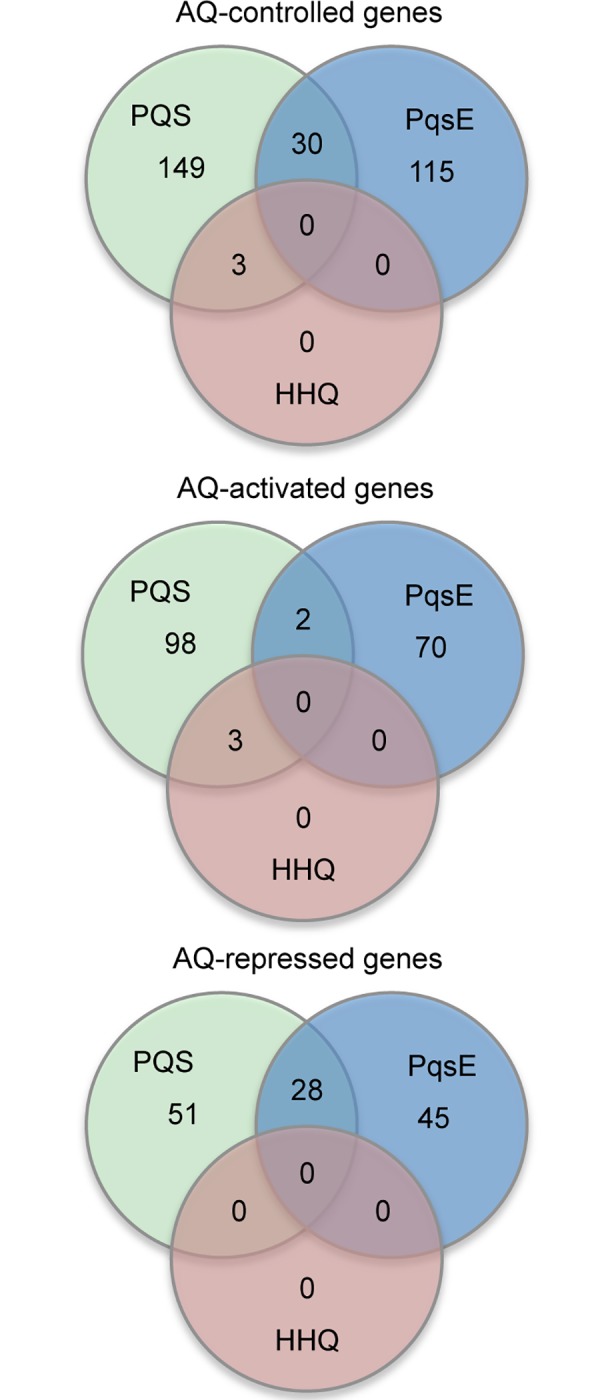
The AQ and PqsE regulons. Venn diagrams showing the number of genes controlled by HHQ, PQS, and PqsE in *P*. *aeruginosa* Δ4AQ, and the overlap between the regulons.

Following statistical validation of the dataset, only genes with a fold change > 2.5 and a *q*-value < 0.05 were considered for further analysis [34]. **Table 1** lists the selected genes (see **S1 Table** for complete list) satisfying this cut-off and hence significantly controlled by the AQs and/or by PqsE. In brief, the RNA levels for 0, 3, 145, and 182 genes were significantly altered by HQNO, HHQ, PqsE and PQS respectively (**Fig 2**).

**Table 1:** Selected genes whose transcription is controlled by HHQ, PQS and/or PqsE.

PA number ^a^	Gene name ^a^	HHQ ^b^	PQS ^c^	PqsE ^d^	Product name ^a^
PA0051	*phzH*			3.2 (3.6)	Potential phenazine-modifying enzyme
PA0083	*tssB1*			-5.1(-1.4)	TssB1
PA0084	*tssC1*			-3.5 (-1.3)	TssC1
PA0263	*hcpC*		-4.3		Secreted protein Hcp
PA0997* ^∫^ ^‡^ ^◊^	*pqsB*	6.7	17.5		PqsB
PA0998* ^∫^ ^‡^ ^◊^	*pqsC*	5.5	16.1		PqsC
PA0999* ^∫^ ^‡^ ^◊^	*pqsD*	5.8	15.7		3-oxoacyl-[acyl-carrier-protein] synthase III
PA1000* ^∫^ ^◊^	*pqsE*			22.8 (140.3)	Quinolone signal response protein
PA1001* ^∫^ ^◊^	*phnA*			26.2 (ncd)	Anthranilate synthase component I
PA1002* ^∫^ ^◊^	*phnB*			22.4 (ncd)	Anthranilate synthase component II
**PA1245** ^§^ ^∫^	***aprX***		3.9		AprX
PA1706	*pcrV*		2.7		Type III secretion protein PcrV
PA1707	*pcrH*		3.1		Regulatory protein PcrH
PA1708	*popB*		5.6		Translocator protein PopB
PA1709	*popD*		3.0		Translocator outer membrane protein PopD precursor
PA1710	*exsC*		3.5		ExsC exoenzyme S synthesis protein C precursor
PA1711	*exsE*		3.1		ExsE
PA1712	*exsB*		2.6		Exoenzyme S synthesis protein B
PA1718	*pscE*		4.3		Type III export protein PscE
PA1901^∫^	*phzC2*			5.5 (6.3)	Phenazine biosynthesis protein PhzC
PA1902^§^	*phzD2*			7.5 (9.8)	Phenazine biosynthesis protein PhzD
PA1903^∫^	*phzE2*			8.8 (9.6)	Phenazine biosynthesis protein PhzE
PA1904	*phzF2*			10.3 (9.9)	Probable phenazine biosynthesis protein
PA1905	*phzG2*			9.7 (9.6)	Probable pyridoxamine 5'-phosphate oxidase
PA2193*	*hcnA*			3.6 (2.1)	Hydrogen cyanide synthase HcnA
PA2194*	*hcnB*			3.1 (1.7)	Hydrogen cyanide synthase HcnB
PA2195*	*hcnC*			3.0 (1.6)	Hydrogen cyanide synthase HcnC
PA2300* ^∫^ ^◊^	*chiC*			18.7 (8.2)	Chitinase
**PA2426**	***pvdS***		43.9		Sigma factor PvdS
PA2570* ^∫^ ^◊^	*lecA *			26.3 (15.1)	LecA lectin
PA3361* ^◊^	*lecB*			8.5 (10.4)	Fucose-binding lectin LecB
PA3391	*nosR*		-4.6	-4.1	Regulatory protein NosR
PA3478*	*rhlB*			3.6 (2.3)	Rhamnosyltransferase chain B
PA3479	*rhlA*			3.6 (2.3)	Rhamnosyltransferase chain A
PA3841	*exoS*		2.6		Exoenzyme S
PA3842	*spcS*		3.9		Specific *Pseudomonas* chaperone for ExoS, SpcS
**PA4175**	***prpL***		3.7		PrpL, protease IV
PA4205* ^∫^ ^◊^	*mexG*			25.0 (47.2)	Hypothetical protein
PA4206* ^∫^ ^◊^	*mexH*			16.4 (28.8)	Probable RND efflux membrane fusion protein precursor
PA4207* ^∫^ ^◊^	*mexI*			18.5 (18.2)	Probable RND efflux transporter
PA4208* ^∫^ ^◊^	*opmD*			11.6 (9.3)	Probable outer membrane protein precursor
PA4209* ^∫^ ^◊^	*phzM*			4.1 (8.7)	Probable phenazine-specific methyltransferase
PA4210^◊^	*phzA1*			10.2 (16.2)	Probable phenazine biosynthesis protein
PA4211* ^◊^	*phzB1*			5.6 (10.1)	Probable phenazine biosynthesis protein
PA4217* ^§^ ^◊^	*phzS*			9.0 (9.3)	Flavin-containing monooxygenase
**PA4227** ^∫^ ^‡^	***pchR***		11.2		Transcriptional regulator PchR
**PA4468** ^§^	***sodA***		89.4		Superoxide dismutase
**PA4470** ^§^ ^∫^	***fumC1***		114.7		Fumarate hydratase
PA4648	*cupE1*			3.0 (2.2)	Pilin subunit CupE1

^a^ PA number, gene name and product name are from the *Pseudomonas* Genome Database [13]. Genes previously reported as controlled by iron-starvation are in bold characters [40,41].

*, genes whose transcription was altered in the ∆*pqsR* mutant with respect to the wild type strain [10]

§, genes whose transcription was altered upon exogenous PQS provision [28]

∫, genes whose transcription was altered in the ∆*pqsA* mutant with respect to the wild type strain [11]

‡, genes whose transcription was altered upon PqsE overexpression [11]

◊, genes whose transcription was altered in the ∆*pqsH* mutant with respect to the wild type strain [23]. RND, Resistance-Nodulation-Cell division; MFS, major facilitator superfamily.

^b^ Fold change in gene expression in *P*. *aeruginosa* PAO1 ∆4AQ grown in the LB supplemented with 40 μM HHQ with respect to the same strain grown in LB.

^c^ Fold change in gene expression in *P*. *aeruginosa* PAO1 ∆4AQ grown in the LB supplemented with 40 μM PQS with respect to the same strain grown in LB.

^d^ Fold change in gene expression in *P*. *aeruginosa* PAO1 ∆4AQ grown in the LB supplemented with 1 mM IPTG (to induce PqsE expression) with respect to the same strain grown in LB; in brackets is indicates the fold change in gene expression in *P*. *aeruginosa* PAO1 ∆*pqsAHLE* pUCP*pqsE* with respect to *P*. *aeruginosa* PAO1 ∆*pqsAHLE* pUCP18, both grown in LB. For the microarray analysis performed in the ∆*pqsAHLE* background, fold changes with a *q* value < 0.05 are indicated for selected virulence related genes, irrespective of the fold change. ncd, no change detected (*q* value > 0.05).

HQNO had no effect on the *P*. *aeruginosa* ∆4AQ transcriptome indicating that this AQ does not function as a QS signal, implying an alternative role for HQNO in *P*. *aeruginosa* physiology. Since HQNO is a potent cytochrome *bc*_*1*_ complex inhibitor [9], it is likely that HQNO acts primarily as a secondary metabolite that increases the environmental competitiveness of *P*. *aeruginosa*.

Notably, only 3 genes were significantly controlled by HHQ, namely *pqsB*, *pqsC* and *pqsD* (**Table 1**; **Fig 2**). This suggests that HHQ controls only the *pqsABCDE* transcriptional unit so driving the positive feedback loop. The positive effect of HHQ on P*pqsA* activity is mediated by PqsR [22,24], such that the primary role of HHQ as a signal is to induce the PqsR-dependent expression of the *pqsABCDE* transcriptional unit, ultimately resulting in increased AQ biosynthesis and *pqsE* expression. As expected, the *pqsB*, *pqsC* and *pqsD* genes were also identified among the genes up-regulated by PQS (**Table 1**).

In the Δ4AQ mutant background, PqsE emerges as a major effector of the *pqs* QS system, since the microarray analysis revealed it controls the expression of 145 genes in the ∆4AQ strain, an AQ-negative background in which PqsE is unable to down-regulate AQ production. In particular, 72 genes were up-regulated and 73 down-regulated upon IPTG-induction of *pqsE* expression (**Fig 2**; **S1 Table**).

The 72 genes up-regulated by *pqsE* expression, included the pyocyanin biosynthetic genes (*phzA*, *phzB*, *phzC*, *phzD*, *phzE*, *phzF*, *phzG*, *phzM*, *phzS*), the *hcnABC* operon required for hydrogen cyanide biosynthesis, *rhlA* and *rhlB*, required for rhamnolipid biosynthesis, and *chiC*, coding for the extracellular chitinase ChiC. Moreover, PqsE exerted a positive effect on the transcription of genes involved in biofilm development *e*.*g*. *cupE1*, *lecA* and *lecB*, explaining the positive control of PqsE on biofilm formation [11], and on the *mexGHI-opmD* operon, coding for a Resistance-Nodulation-Cell division (RND) efflux pump involved in antibiotic resistance that is also essential for *pqs*-dependent QS. This is because *mexG* and *opmD* mutants are both avirulent in plant and rat infection models and fail to produce PQS, probably as a consequence of the intracellular accumulation of a toxic AQ metabolite [35]. Furthermore, pyocyanin functions as a signal in the *P*. *aeruginosa* QS network because it induced changes in the expression of over 50 genes (23 up-regulated and 29 down-regulated) [36]. Of these only *mexGHI-opmD*, PA2274 and PA3250 were also up-regulated *via* PqsE rather than PQS (**Tables 1** and **S1**). Hence, although PqsE controls pyocyanin biosynthesis, it only regulates a sub-set of pyocyanin-dependent genes.

**Fig 3:**
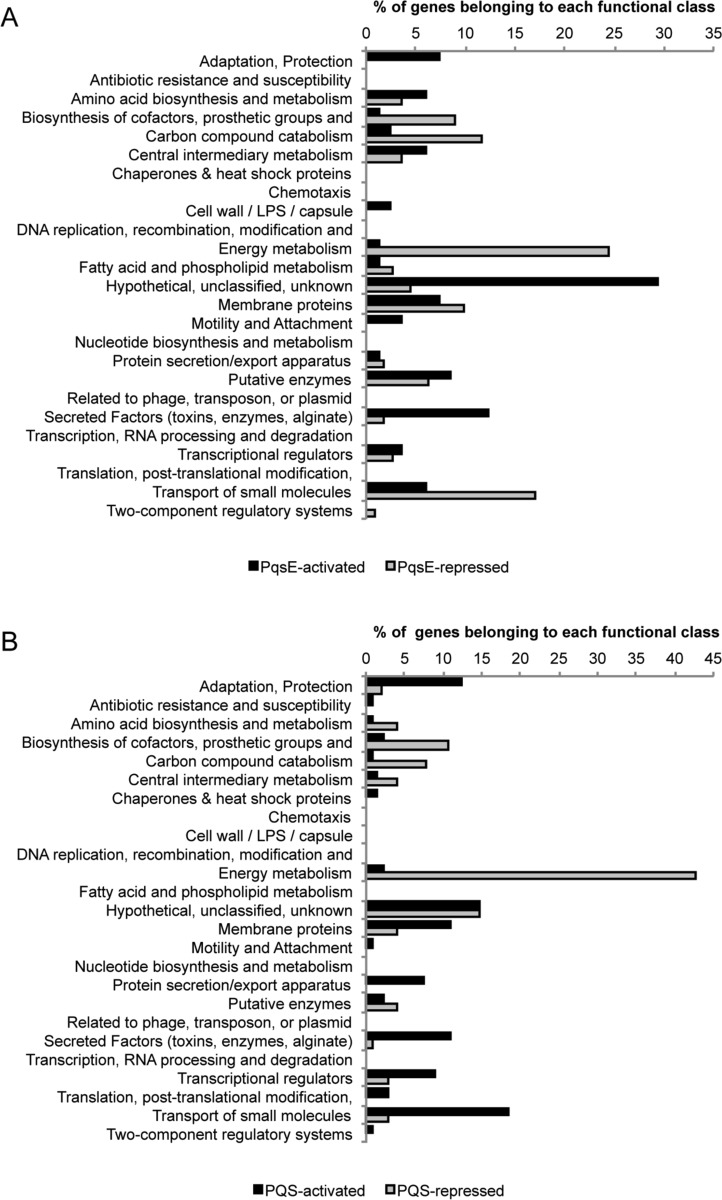
Functional classes of PqsE and PQS controlled genes. Histograms representing the distribution of (**A**) PqsE-controlled and (**B**) PQS-controlled genes according to their functional classification. Functional classes are from the *Pseudomonas* Genome Database [13].

As shown in **Fig 3A**, many of PqsE up-regulated genes belong to the “Secreted Factors (toxins, enzymes, alginate)” and “Adaptation, Protection” functional classes (12.2% and 7.3%, respectively), highlighting the importance of PqsE in *P*. *aeruginosa* adaptive behaviour and virulence. However, most of the PqsE up-regulated genes (29.3%) are classified as “Hypothetical, unclassified, unknown”, limiting our comprehension of its physiological role.

With respect to the 73 genes down-regulated upon *pqsE* induction, they are mainly involved in energy metabolism and anaerobic respiration, including *gapA*, coding for glyceraldehyde 3-phosphate dehydrogenase, and almost all the *nir*, *nor*, *nar*, and *nos* genes (**S1 Table**). Indeed, the majority of the PqsE-repressed genes cluster in the “Energy metabolism” (24.3%), “Transport of small molecules” (17.1%), “Carbon compound catabolism” (11.7%) and “Biosynthesis of cofactors, prosthetic groups, and carriers” (9.0%) functional classes (**Fig 3A**). However, the physiological relevance of this repression is not clear, since the IPTG-mediated induction of PqsE does not affect bacterial growth, at least under aerobic conditions. It is also noticeable that two genes involved in type 6 secretion (T6SS; *tssB1* and *tssC1*) are down-regulated in response to *pqsE* induction (**Table 1**).

The global effect exerted by PqsE on the *P*. *aeruginosa* transcriptome is unlikely to be direct, since this protein does not possess a DNA-binding domain [37]. Moreover, PqsE activity is not exclusively a consequence of its thioesterase activity since *pqsE* expression is sufficient to restore pyocyanin in an AQ-deficient (*pqsA* mutant) background [6,11]. Hence, the multifunctional activity of PqsE may conceivably be a consequence of a *pqsE* regulatory RNA acting on the expression of *pqsE*-controlled genes. To investigate this possibility we quantified pyocyanin production in *P*. *aeruginosa* PAO1 ∆*pqsA* ∆*pqsE* double mutant strains carrying plasmids for IPTG-inducible expression of wild type *pqsE*, or *pqsE* mutated variants lacking the first two codons (*pqsE*∆1–6) or with a nucleotide insertion after the ATG to alter the protein frame (*pqsE*NoFrame). As shown in **S2A Fig**, pyocyanin production in the *P*. *aeruginosa* PAO1 ∆*pqsA* ∆*pqsE* strains was restored only upon complementation with wild type *pqsE*, despite the presence of a *pqsE* transcript in the mutated variants (**S2B Fig**). These data suggest that the activity is not due to the *pqsE* RNA transcript but requires the PqsE protein, a finding that suggests PqsE has independent regulatory and thioesterase enzymatic functions.

When the ∆4AQ strain was grown with IPTG, the *phnAB* operon was also up-regulated (**Table 1**). Knoten and co-workers reported that when *P*. *aeruginosa* PAO1 was grown in nutrient limiting conditions but not in LB, *pqsE* and *phnAB* were co-transcribed [38]. However, RT-PCR analysis of the PAO1 strain used in this study indicates that *pqsE* and *phnAB* are co-transcribed after growth in LB (**S3 Fig**), a finding consistent with the fold change increases quantified for *pqsE* (22.8), *phnA* (26.2) and *phnB* (22.4) upon addition of IPTG to the ∆4AQ strain (**Table 1**). Although anthranilate is an AQ precursor [15], *pqsE*-overexpression results in the abrogation of AQ production *via* the strong repression of P*pqsA* promoter [11]. This repression is not apparent in our microarray analysis as a consequence of the lack of HHQ- and PQS-dependent P*pqsA* activation in the ∆4AQ strain.

Anthranilate for AQ biosynthesis can be supplied by the anthranilate synthases TrpEG and PhnAB or *via* the kynurenine pathway that converts tryptophan into anthranilate [38]. The latter is the main source of anthranilate for PQS biosynthesis when tryptophan is present, and PhnAB appears to supply anthranilate only under nutrient-limiting conditions [38]. Consequently the increased transcription of *phnAB* following the IPTG-dependent induction of *pqsE* in the ∆4AQ strain may increase intracellular anthranilate levels and so impact on gene expression independent of PqsE. To explore this possibility, we first quantified intracellular anthranilate levels in the ∆4AQ strain grown in LB or in LB supplemented with 1 mM IPTG. **S4A Fig** shows that the IPTG-induced increase in *phnAB* expression did not result in higher levels of intracellular anthranilate. The slightly higher concentration of anthranilate in the ∆4AQ strain in the absence of IPTG may be a consequence of the PqsE-mediated increases in the expression of genes such as *antR* and *catB* that are involved in the degradation of anthranilate (**S1 Table**). To confirm these data, we also compared the transcriptional profiles of a *P*. *aeruginosa* PAO1 quadruple mutant strain with deletions in *pqsA*, *pqsH*, *pqsL* and *pqsE* (∆*pqsAHLE*) and carrying a plasmid-borne copy of *pqsE* or the empty vector (pUCP18). The data obtained for selected virulence related genes are summarized in **Table 1**. The plasmid-mediated expression of *pqsE* in the ∆*pqsAHLE* genetic background did not affect *phnAB* expression. In addition, the data obtained was broadly consistent with that obtained for the inducible *pqsE* construct with respect to the genes involved in virulence factor production, biofilm formation and antibiotic resistance (Table 1). In addition, we validated the data with respect to the pyocyanin biosynthetic genes by introducing P*phzA1*::*lux* and P*phzA2*::*lux* transcriptional fusions onto the chromosome of the ∆*pqsAHLE* strain, since the microarray experiments cannot discriminate between the two *phz* operons as they are almost identical at the DNA level [39]. The results obtained with the reporter fusions confirm the microarray data and reveal that PqsE is responsible for driving the expression of *phzA1* but not *phzA2* (**S4B Fig**).

Despite the structural similarity between HHQ and PQS and their ability to activate PqsR *via* the same ligand binding site [24], the microarray data revealed that, in contrast to HHQ, PQS regulates the expression of 182 genes. In particular, 103 genes were up-regulated and 79 genes were down-regulated in response to exogenous PQS (**Fig 2**; **S1 Table**). The major proportion of PQS up-regulated genes (75%) are also induced by iron-starvation [40,41]. These consist of almost all the genes involved in the biosynthesis, uptake and response to the siderophores pyoverdine and pyochelin, including the regulatory genes *pvdS* and *pchR*. Moreover, metabolic and virulence genes previously shown to be induced by iron-starvation were strongly up-regulated by PQS, including fumarate hydratase (*fumC1*), superoxide dismutase (*sodA*) and two proteases (*prpL* and *aprX*) (**Table 1**). These findings are consistent with the iron-chelating activity of PQS inducing an iron-starvation response [27,28]. In addition to the iron-regulated genes, PQS increased the transcription of genes involved in Type 3 secretion (T3S; *pcrV*, *pcrH*, *popB*, *popD*, *exsC*, *exsE*, *exsB*, and *pscE*), and coding for both exotoxin ExoS (*exoS*) and its chaperone SpcS (*spcS*), indicating that PQS, independent of PqsE, contributes to *P*. *aeruginosa* virulence gene regulation (**Table 1**). PQS production has been indirectly linked to the regulation of T3S effector secretion in *P*. *aeruginosa* at the post-transcriptional level [42]. Interestingly, the *rhl* QS system that represses both *pqsA* and *pqsR* also negatively regulates *exoS* [43]. As anticipated, *pqsB*, *pqsC* and *pqsD* genes were all up-regulated by PQS (**Table 1**).

The 79 PQS-repressed genes mainly cluster in the “Energy metabolism” (42.7%), “Biosynthesis of cofactors, prosthetic groups, and carriers” (10.7%), and “Carbon compound catabolism” (7.8%) functional classes (**Fig 3B**). The repression exerted by PQS on certain metabolic genes could be due to its interaction with membranes and consequent perturbation of associated energy generation. Almost all the genes involved in denitrification (*nir*, *nor*, *nar*, and *nos* genes) are also down-regulated by PQS (**S1 Table**). These data are in line with previous work demonstrating that PQS represses anaerobic growth of *P*. *aeruginosa* by inhibiting denitrifying enzymes [44].

A comparison of the genes regulated by PQS and PqsE revealed that they control quite distinct regulons and up-regulate different sets of virulence genes (**Table 1**). The PQS and PqsE regulons only share 30 genes (**Fig 2**). Notably, 28 genes independently down-regulated by PQS and PqsE are all involved in denitrification (*nir*, *nor*, *nar*, and *nos* genes; **S1 Table**), indicating that there is some redundancy in the *pqs* system.

**Fig 4:**
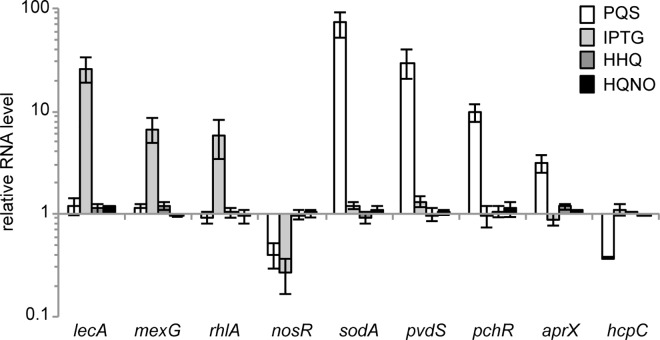
Validation of the microarray data by Real Time PCR. Relative mRNA levels of the genes indicated quantified by Real Time PCR in the *P*. *aeruginosa* ∆4AQ strain grown in LB supplemented with 1 mM IPTG to induce PqsE expression (light-grey bars), or with 40 μM PQS (white bars), HHQ (dark-grey bars), or HQNO (black bars), with respect to the same strain grown in LB. The average of two independent analyses each performed on three technical replicates is shown with standard deviations.

The reliability of the microarray data is supported by the observation that HHQ controls only one transcriptional unit, HQNO does not affect transcription under the growth conditions employed whereas PQS regulates 182 genes. Differential expression of selected genes by PQS or PqsE in the microarray experiment was validated by Real Time PCR analysis. A comparison between **Table 1** and **Fig 4** shows that the results obtained match the microarray data, since the mRNA levels of the *lecA*, *mexG* and *rhlA* genes increased upon IPTG-dependent induction of PqsE, while *nosR* decreased. Similarly, PQS increased the transcription of *sodA*, *pvdS*, *pchR*, and *aprX* but repressed *nosR* and *hcpC*. PqsE did not affect the transcript levels of PQS-controlled genes (*i*.*e*., *sodA*, *pvdS*, *pchR*, *aprX* and *hcpC*), and conversely PQS did not alter PqsE-regulated transcript levels (*i*.*e*., *lecA*, *mexG* and *rhlA*). Moreover, none of these transcripts were affected by HHQ or HQNO (**Fig 4**).

The potential effects of the AQs and/or PqsE on *pqsH* or *pqsL* transcription cannot be inferred from the microarray analysis, since both genes were deleted in the ∆4AQ strain. Therefore, the expression of chromosomal *pqsH* and *pqsL lux* promoter fusions was investigated in the *P*. *aeruginosa* ∆4AQ strain. Neither promoter was influenced by exogenous HHQ, PQS or HQNO or by *pqsE* induction (**S5 Fig**). Thus, the autoregulatory activity of PQS is not directly exerted at the level of *pqsH* transcription, and HQNO has no effect in promoting its own biosynthesis. These data imply that a positive feedback loop exists in the *pqs* QS system only at the level of the *pqsABCDE-phnAB* transcriptional unit, with HHQ and PQS promoting their own biosynthesis by inducing P*pqsA* activity *via* PqsR.

Overall, our microarray experiments are consistent with previously published transcriptomic analyses highlighting the contributions of the *pqs* system to virulence factor production, ferric iron acquisition and energy metabolism [6,10,11,23,28]. However, our approach enabled us to discriminate between the physiological roles played by the distinct elements of the *pqs* QS system. For example, both PQS and PqsE were reported to affect iron-controlled genes, probably because in previous experimental settings, PqsE could control AQ biosynthesis [6,11]. However, our data demonstrate clearly that PQS but not PqsE, regulates the iron-regulated genes. Similarly, certain PqsE-controlled virulence factors (*e*.*g*., pyocyanin, lectins, ChiC chitinase and the MexGHI-OpmD efflux pump) were reported to be PQS-controlled, probably because in previous experiments the addition of synthetic PQS or the abrogation of PQS synthesis (in *pqsR*, *pqsA* or *pqsH* mutants) led to dysregulation of *pqsE* expression [10,23,28]. Moreover, the use of the ∆4AQ strain provides clear evidence that HQNO does not influence the *P*. *aeruginosa* transcriptome, and that HHQ exclusively regulates the *pqsABCDE-phnAB* transcriptional unit. Therefore, HHQ activity ultimately leads to the indirect control of specific physiological processes by increasing the expression of the effector protein PqsE and by acting as a substrate for PQS biosynthesis.

### Comparison of the PQS and PqsR regulons reveals a PqsR-independent PQS regulon

Despite the structural similarity of the two AQs, HHQ controls the transcription of a single transcriptional unit (*i*.*e*., *pqsABCDE-phnAB*), while PQS regulates 182 genes. Since both AQs act as PqsR co-inducers [22,24], it is possible that the PqsR-HHQ complex only affects P*pqsA* activity, while the PqsR-PQS complex acts more globally as do other QS regulators such as LasR. However, PQS appears to influence gene expression *via* PqsR-dependent and PqsR-independent mechanisms, for example, by inducing an iron-starvation response [27,28].

To discriminate between PqsR-dependent and PqsR-independent PQS regulons, and to characterize the PqsR regulon itself, *pqsR* was deleted in the ∆4AQ strain, generating the quintuple *P*. *aeruginosa* ∆5AQ mutant (**Fig 1**). The transcriptomes of the ∆4AQ and ∆5AQ mutants, supplemented with PQS (40 μM), were compared. Only 4 genes were significantly down-regulated in the ∆5AQ strain with respect to the ∆4AQ mutant, namely *pqsR*, *pqsB*, *pqsC*, and *pqsD* (-101.2 < fold change < -186.2). An apparent strong down-regulation of *pqsR* was expected since this gene has been deleted from *P*. *aeruginosa* ∆5AQ. The down-regulation of *pqsB*, *pqsC*, and *pqsD* in *P*. *aeruginosa* ∆5AQ strongly suggests that in PAO1 PqsR only triggers the transcription of the *pqsABCDE*-*phnAB* operon, and thus the *pqsA* promoter region is the only target for the PqsR-PQS complex.

Overall, these data imply that, apart from the *pqs* genes, the other 179 genes identified as PQS-regulated (**S1 Table**) are controlled by PQS *via* a PqsR-independent pathway(s). This regulatory activity is likely due, at least in part, to the iron-chelating activity of PQS, consistent with the finding that 77/100 genes up-regulated by PQS in a PqsR-independent manner are known to be induced by iron-starvation [40,41]. In contrast the 79 genes down-regulated by PQS have not previously been reported to be repressed in low-iron media. PQS could conceivably also control other phenotypes in both an iron- and a PqsR-independent manner through direct interactions with the outer membrane [25] or by acting as a pro- or anti-oxidant [45].

### PQS activates P*pqsA via* both PqsR-dependent and PqsR-independent pathways

The transcriptome analysis performed on the ∆4AQ strain indicates that PQS is more potent than HHQ in activating transcription of the *pqsABCDE-phnAB* operon in LB medium (**Table 1**). This is also consistent with a chromosomal reporter P*pqsA*::*lux* fusion in a *P*. *aeruginosa pqsAH* mutant, which cannot convert exogenously supplied HHQ to PQS, where EC_50_ values of 16.4±2.6 μM and 3.8±1.6 μM for HHQ and PQS respectively have been determined in LB medium [24]. However, HHQ activates the P*pqsA* promoter at a similar level to PQS when the bacteria are grown in an iron-deficient casamino acids (CAA) medium [27]. This suggests that PQS is more effective than HHQ in stimulating P*pqsA* activity because it induces an iron-starvation response. This hypothesis would be in agreement with iron-chelating activity of PQS, with the evidence that high-iron concentrations negatively impact on P*pqsA* activity [6].

To investigate this possibility, the effect of HHQ, PQS and 2-heptyl-3-amino-4-quinolone (3-NH_2_-PQS) on both P*pqsA* and P*pqsR* activation were compared in the ∆4AQ (*pqsR*-proficient) and ∆5AQ (*pqsR*-mutant) strains, grown in LB with or without 100 μM FeCl_3_. 3-NH_2_-PQS is a potent PqsR agonist (EC_50_ 0.4±0.1 μM) isosteric with PQS but lacking iron-chelating activity [24]. In addition, although *pqsR* was not identified among the AQ-controlled genes in the transcriptome analysis (**S1 Table**) we included the *pqsR* promoter fusion experiments since it is not possible to exclude a regulatory effect below the fold change cut-off used (> 2.5) that has a significant effect on P*pqsA* activity.

**Fig 5:**
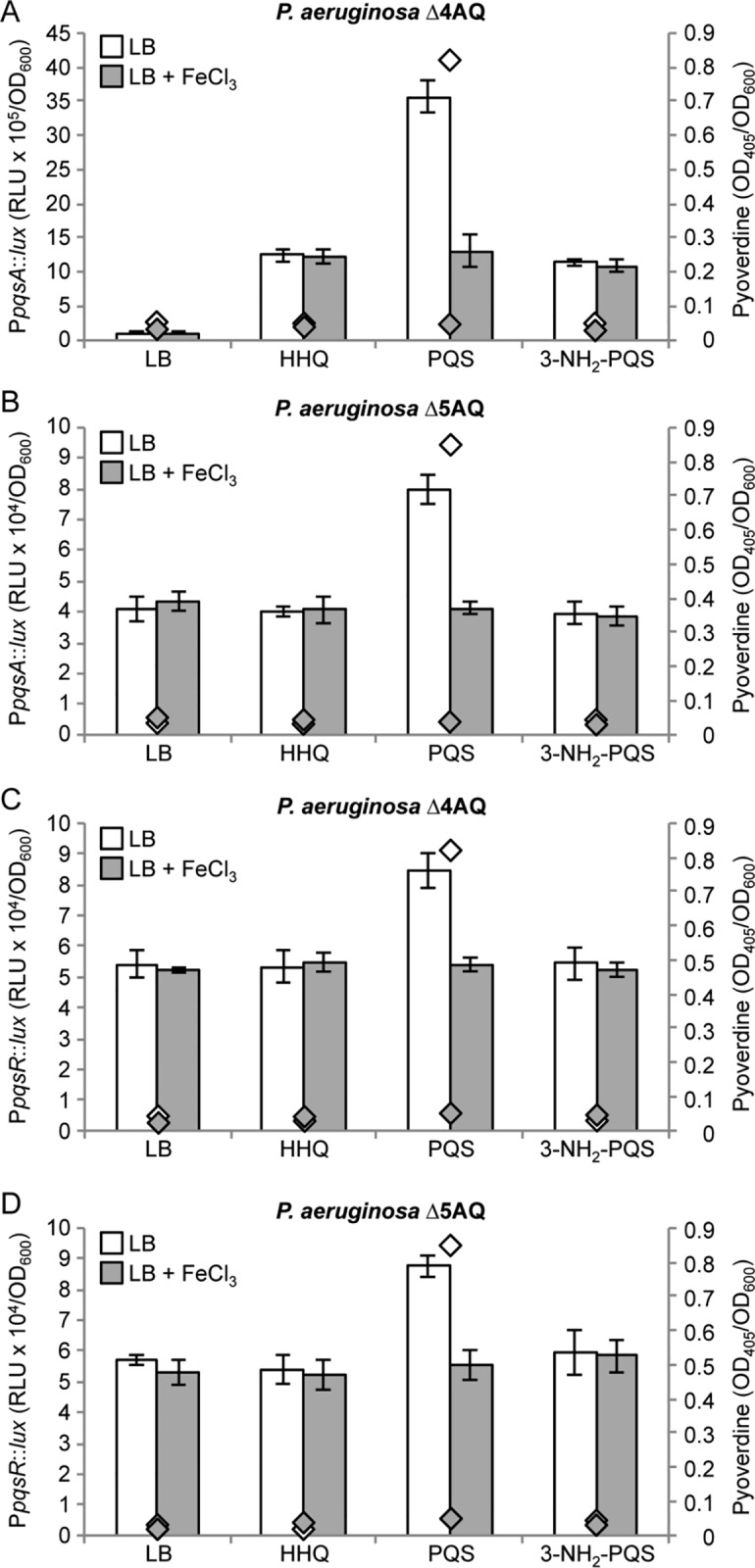
Interplay of HHQ, PQS, PqsR and iron in controlling P*pqsA* and P*pqsR* activity. Maximal promoter activity quantified in the indicated strains carrying the transcriptional fusions P*pqsA*::*lux* (**A** and **B**) or P*pqsR*::*lux* (**C** and **D**). Strains were grown in LB or in LB supplemented with 40 μM HHQ, PQS or 3-NH_2_-PQS, as indicated below the graphs, in the absence (white bars) or presence (grey bars) of 100 μM FeCl_3_. Diamonds indicate the pyoverdine levels in the absence (white diamonds) or in the presence (grey diamonds) of 100 μM FeCl_3_. Promoter activity and pyoverdine level are reported as Relative Light Units (RLU) and OD_405_, respectively, normalized to cell density (OD_600_). The average of three independent experiments is reported with standard deviations.

Consistent with previous work, PQS was more effective than HHQ in up-regulating P*pqsA* in LB, while HHQ and 3-NH_2_-PQS induced P*pqsA* activity at similar levels (**Fig 5A**). HHQ and 3-NH_2_-PQS did not induce P*pqsA* in the *pqsR* mutant strain ∆5AQ, while PQS exerted a positive, ∼2 fold induction of P*pqsA* in this genetic background (**Fig 5B**). HHQ and 3-NH_2_-PQS had no effect on P*pqsR* activity, while P*pqsR* was induced by PQS in both the ∆4AQ and ∆5AQ strains (~1.5 fold; **Fig 5C and 5D**). Interestingly, the PQS-dependent induction of P*pqsA* in the ∆4AQ strain was strongly reduced when iron was added to the medium, showing the same activation level as that induced by HHQ and 3-NH_2_-PQS. Iron supplementation had no effect on P*pqsA* activity when the promoter was induced with HHQ or 3-NH_2_-PQS (**Fig 5A**). The reduced ability of PQS to induce P*pqsA* activity in the presence of 100 μM FeCl_3_ is likely due to its inability to induce P*pqsA* and P*pqsR via* the PqsR-independent pathway in the presence of high iron concentrations (**Fig 5B–5D**).

A plausible explanation for the above results is that the iron-chelating activity of PQS decreases the levels of available iron in LB medium, triggering an iron-starvation response with consequent activation of P*pqsA* and P*pqsR via* a PqsR-independent pathway. Indeed, the siderophore pyoverdine, which is only produced under iron limiting conditions [46], is detectable when PQS is added to the ∆4AQ and ∆5AQ cultures, but not when PQS is replaced with HHQ or 3-NH_2_-PQS, or by excess iron (**Fig 5**), confirming that PQS triggers an iron-starvation response in LB. This is also in line with increased expression of the Fur-controlled iron-starvation sigma factor PvdS in the presence of PQS (**Table 1**).

### The PqsR-independent activation exerted by PQS on P*pqsA* and P*pqsR* is not mediated by the iron-starvation response pathway

To determine whether the ability of PQS to induce the P*pqsA* and P*pqsR* promoters *via* a PqsR-independent pathway simply relies on its iron chelating properties, the effects of PQS and the iron chelators 2,2’-dipyridyl and deferiprone on P*pqsA* and P*pqsR* activity were compared in the *P*. *aeruginosa* strains ∆4AQ and ∆5AQ by means of transcriptional fusions. 2,2’-Dipyridyl chelates ferrous iron (Fe^2+^) [47], which is the prevalent intracellular iron species, while deferiprone chelates ferric iron (Fe^3+^), which prevails in extracellular environment, to form a 3:1 (deferiprone:Fe^3+^) complex [48], similar to the 3:1 ferric complexes formed by 2-hydroxy-3-alkyl-4-quinolones such as PQS [27]. Both 2,2’-dipyridyl and deferiprone induce iron-starvation in *P*. *aeruginosa* [49,50]. The results obtained show that 40 μM PQS, 500 μM 2,2’-dipyridyl or 160 μM deferiprone all triggered similar levels of pyoverdine production in LB-grown cultures. However, neither 2,2’-dipyridyl nor deferiprone induce P*pqsA* or P*pqsR* activity, irrespective of the presence of PqsR (**S6 Fig**). These data strongly suggest that the PqsR-independent effect exerted by PQS on P*pqsA* and P*pqsR* does not depend on the ability of PQS to induce an iron-starvation response. Consistent with these findings, the activity of the P*pqsA* and P*pqsR* promoters was unchanged in the 4AQ and ∆5AQ strains upon mutation of the *pvdS* gene that codes for the iron-starvation response sigma factor PvdS (**S7A–S7D Fig**); no differences in P*pqsA* and P*pqsR* activity were observed when these promoters were tested in *P*. *aeruginosa* PAO1 wild type and its isogenic ∆*pvdS* mutant (**S7E Fig**). Moreover, a Fur Titration Assay (FurTA) [51] revealed that the iron-response regulator Fur does not bind to the P*pqsA* or P*pqsR* promoter regions (**S7F Fig**).

**Fig 6:**
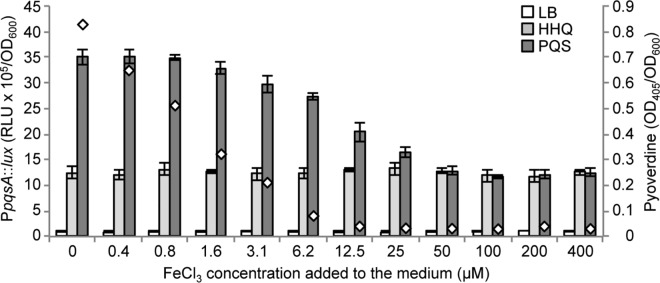
Effect of iron on the ability of PQS to stimulate P*pqsA* activity. Maximal P*pqsA* promoter activity measured in the *P*. *aeruginosa* ∆4AQ strain carrying the transcriptional fusion P*pqsA*::*lux*, grown in LB (white bars) or in LB supplemented with 40 μM HHQ (light-grey bars) or 40 μM PQS (dark-grey bars), and FeCl_3_ at the concentration indicated below the graph. White diamonds indicate the pyoverdine level in the supernatants of cultures grown in the presence of PQS with or without FeCl_3_. Promoter activity and pyoverdine are reported as Relative Light Units (RLU) and OD_405_, respectively, normalized to cell density (OD_600_). The average of three independent experiments is reported with standard deviation.

Collectively, these data demonstrate that the ability of PQS to induce P*pqsA* and P*pqsR via* a PqsR-independent pathway does not rely on the capacity of PQS to induce an iron-starvation response *via* the master regulators PvdS and Fur. However, the effect of PQS on both promoters is inhibited when 100 μM FeCl_3_ is added to the LB medium (**Fig 5**). To clarify this finding further, the ability of PQS to induce P*pqsA* activity was determined in *P*. *aeruginosa* ∆4AQ grown in LB supplemented with increasing concentrations of FeCl_3_ without AQs or with either PQS or HHQ. In parallel, pyoverdine levels were determined in the culture supernatants of the ∆4AQ strain grown in the presence of PQS to monitor the activation of the iron-starvation response. As shown in **Fig 6**, iron had no effect on the ability of HHQ to induce P*pqsA* activity or on the P*pqsA* basal level in the absence of AQs. Conversely, increasing concentrations of FeCl_3_ reduced the ability of PQS to promote P*pqsA* activity, and inhibited pyoverdine production, consistent with our previous data. Low FeCl_3_ concentrations (from 0.4 μM to 1.6 μM) were sufficient to decrease the iron-starvation response, as indicated by reduced pyoverdine production, without affecting the PQS-dependent induction of P*pqsA*. The ability of HHQ and PQS to induce P*pqsA* was comparable when the medium iron concentration approximated to the theoretical PQS-saturating value, ranging from 12.5 to 25 μM (considering 40 μM PQS and 3:1 ratio of the PQS-Fe^3+^ complex).

Given that iron reduces the PqsR-independent expression of the *pqsA* or *pqsR* promoters in the absence of PvdS and that Fur does not bind to either promoter, our data suggest a regulatory role for PQS in the absence of PqsR. This regulatory activity can however be abolished by increasing the medium iron content. However, of the 179 genes regulated by PQS *via* PqsR-independent pathways only 77 are controlled by the iron-starvation response. The remaining 102 genes (underlined in **S1 Table**) are regulated *via* an iron-starvation-independent and PqsR-independent PQS signalling pathway(s). Most of the repressed genes are involved in energy metabolism (49%) and include the *nir*, *nar*, *nos* genes involved in denitrification. Of the up-regulated genes, 39% are from the protein secretion/export functional class and include T3S genes such as *pcrV*, *exsC*, *exsE*, *exoS* and *spcS*. The mechanism by which these genes are regulated is not yet apparent but may be due to the anti-oxidant properties of PQS since these are likely to be inhibited by excess iron [45].

Since PQS promotes P*pqsA* activity in *P*. *aeruginosa* at different levels depending on the availability of iron, the expression of PqsE-controlled virulence factors in iron-poor environments is likely to be higher. In this context, it is tempting to speculate that the iron chelating ability of PQS may contribute to *P*. *aeruginosa* environmental fitness both by limiting the availability of iron to competing microorganisms and by increasing the expression of specific sets of genes important in challenging iron-poor environments. This process might be relevant during the colonization of the human host, when *P*. *aeruginosa* experiences iron starvation, and implies a new role for PQS as an extracellular iron sensor.

## Conclusions

**Fig 7:**
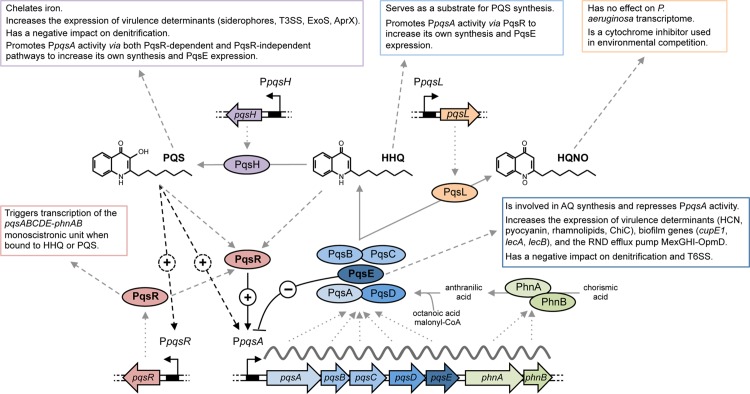
Schematic representation of the *pqs* QS system in *P*. *aeruginosa*. The core of the *pqs* QS system is composed of the *pqsABCDE-phnAB* operon and the *pqsR* gene. Proteins coded by the *pqsABCDE-phnAB* operon synthesize HHQ that binds to and activates PqsR. The PqsR-HHQ complex promotes P*pqsA* activity, thus increasing HHQ and PqsE levels. Notably, the P*pqsA* promoter is the only target of the PqsR-HHQ complex. Apart from its contribution to HHQ biosynthesis, PqsE influences the *P*. *aeruginosa* transcriptome *via* a still uncharacterized AQ-independent pathway(s). In this way, PqsE up-regulates the expression of genes involved in virulence factor production, biofilm development, and antibiotic resistance. Conversely, PqsE down-regulates P*pqsA* activity, AQ production and the expression of genes involved in denitrification and T6SS. The *pqsH* and *pqsL* genes are required for PQS and HQNO biosynthesis, respectively. HQNO did not affect the *P*. *aeruginosa* transcriptome, and probably contributes to environmental competition due to its cytochrome inhibitory activity. PQS chelates iron triggering the iron-starvation response and increasing the transcription of virulence factor genes coding for virulence factors such as pyoverdine, ExoS toxin and AprX protease. Moreover, PQS down-regulates genes involved in denitrification. Most of the regulatory effects exerted by PQS are PqsR-independent, since the PqsR-PQS (or PqsR-HHQ) complex only promotes P*pqsA* activity. However, PQS also increases P*pqsA* and P*pqsR* expression *via* a PqsR-independent pathway(s) that is unrelated to the iron-starvation response, but is inhibited in the presence of high-iron concentrations. Dotted grey arrows indicate gene expression; solid grey arrows represent biosynthesis; solid black arrow indicates PqsR-dependent activation (+); dashed black arrows indicate PqsR-independent activation (+); black T-line indicates negative regulation (-); dashed grey arrows represent information flow.

Although the central role of the *pqs* QS system in the control *P*. *aeruginosa* infection processes has been extensively studied, the precise role played by each individual element of this complex regulatory circuit remained to be defined. Here we have filled this knowledge gap by defining the specific genome-wide regulons for HHQ, PQS and HQNO and for the effector PqsE in the presence and absence of PqsR (**Fig 7**). Of 145 genes regulated *via* PqsE only 30 were co-regulated by PQS (**Fig 2**). Among the key genes controlled by PqsE in the absence of AQs are those coding for the MexGHI-OpmD efflux pump and pyocyanin biosynthesis. Although biochemically PqsE functions as a thioesterase in AQ biosynthesis [17], the thioesterase-independent regulatory mechanism controlling gene expression requiring the PqsE protein remains to be elucidated. A striking feature of our transcriptome data is that signalling function of HHQ is simply to drive the expression of the *pqsABCDE-phnAB* transcriptional unit in a PqsR-dependent manner. These data highlight that unlike LuxR/AHL-based QS systems where the response regulator interacts with the promoters of multiple target genes, PqsR appears to target only one, the *pqsABCDE-phnAB* operon. Furthermore, HQNO, the *N*-oxide of HHQ, does not act as a signal molecule. In contrast to HHQ and HQNO, PQS is clearly a multi-functional molecule that operates *via* multiple PqsR-dependent and PqsR-independent pathways. In this it resembles *N*-(3-oxododecanoyl)homoserine lactone (3OC_12_-HSL), which not only modulates the *P*. *aeruginosa* transcriptome *via* LasR and QscR, but also in the absence of any regulators incorporating an AHL-binding domain [52].

## Materials and Methods

### Bacterial strains and media

The bacterial strains used in this study are listed in **S2 Table**. *E*. *coli* and *P*. *aeruginosa* strains were routinely grown at 37°C in Luria-Bertani (LB) broth with aeration. When required, LB was supplemented with synthetic 40 μM HHQ, PQS, or HQNO, or with 1 mM IPTG. FeCl_3_, 2,2’-dipyridyl and deferiprone were used at the concentrations indicated. AQs including 3-NH_2_-PQS were synthesized as described previously [24]. Unless otherwise stated, antibiotics were added at the following concentrations: *E*. *coli*, 100 μg ml^-1^ ampicillin (Ap), 10 μg ml^-1^ tetracycline (Tc), or 30 μg ml^-1^ chloramphenicol (Cm); *P*. *aeruginosa*, 200 μg ml^-1^ tetracycline (Tc), 375 μg ml^-1^ chloramphenicol (Cm), or 400 μg ml^-1^ carbenicillin (Cb).

### Recombinant DNA techniques

The plasmids and oligonucleotides used are listed in **S2** and **S3 Tables** respectively. Preparation of plasmid DNA, purification of DNA fragments, restrictions, ligations, and transformations in *E*. *coli* DH5α or S17.1λ*pir* competent cells were performed with standard procedures. DNA amplification was by Polymerase Chain Reaction (PCR) using the GoTaq Polymerase (Promega).

### Construction of recombinant strains

The *P*. *aeruginosa* ∆4AQ quadruple mutant strain was constructed by allelic exchange using the suicide vectors pDM4Δ*pqsE*ind [11] and pDM4∆*pqsL* in the double mutant *P*. *aeruginosa* PAO1 ∆*pqsA* ∆*pqsH* [27]. The *P*. *aeruginosa* ∆5AQ quintuple mutant was constructed using the suicide vector pDM4Δ*pqsR* [24] in *P*. *aeruginosa* ∆4AQ. The *P*. *aeruginosa* PAO1 ∆*pqsAHLE* quadruple mutant strain was generated by allelic exchange using the suicide vectors pDM4∆*pqsE* [11] and pDM4∆*pqsL* in the double mutant *P*. *aeruginosa* PAO1 ∆*pqsA* ∆*pqsH* [27]. pDM4∆*pqsL* was constructed by PCR amplifying the upstream and downstream fragments (~500 bp) of *pqsL* from PAO1 using the primers FW*pqsL*UP and RV*pqsL*UP, and FW*pqsL*DOWN and RV*pqsL*DOWN, respectively (**S3 Table**). The same procedures were used to introduce *pvdS* mutations into the *P*. *aeruginosa* wild type, ∆4AQ and ∆5AQ strains. In this case, the *E*. *coli* pEX∆*pvdS* strain [53] was used as donor strain in the conjugation step.

For promoter activity studies, transcriptional fusions between the promoter regions of *pqsH*, *pqsL*, *pqsR*, *phzA1*, *phzA2* and the *luxCDABE* operon were constructed using the miniCTX-*lux* plasmid as previously described [27].

### High-density oligonucleotide microarrays

Total RNA for the high-density oligonucleotide microarray experiments was extracted from 1 ml cultures of *P*. *aeruginosa* 4AQ, 5AQ or ∆*pqsAHLE* carrying the plasmid pUCP18 or pUCP*pqsE*, grown at 37°C with shaking at 200 rpm to an OD_600_ 1.5 in LB or in LB supplemented with 40 μM HHQ, PQS, HQNO, or 1 mM IPTG. Cells were mixed with 2 ml of RNA Protect Bacteria Reagent (Qiagen) the cells lysed and RNA was purified using RNeasy mini-columns (Qiagen), including the on-column DNase I digestion step. In addition, we treated the eluted RNA for 1 h at 37°C with TURBO DNase (0.1 units per μg of RNA; Ambion). DNase I was removed with the RNeasy Column Purification Kit (Qiagen). RNA integrity was monitored by agarose gel electrophoresis, and the absence of contaminating chromosomal DNA was verified by PCR with primers pairs FW*pqsB*-RV*pqsB* and FW16SRT-RV16SRT (**S3 Table**).

Processing of the *P*. *aeruginosa* PAO1 Affymetrix GeneChip and statistical analysis of the dataset were performed at the Lausanne Genomic Technologies Facility, Center for Integrative Genomics, University of Lausanne, Switzerland. For each condition, two different pools of RNA were compared (biological duplicate), each containing RNAs from three independent extractions (technical triplicate). Fold changes > 2.5 with a *q*-value < 0.05 were considered as statistically significant. The *q*-value is the smallest False Discovery Rate (FDR) for which the test can be considered significant [34].

### Reverse transcriptase and Real Time PCR analyses

For reverse transcriptase PCR (RT-PCR) and Real Time PCR analyses, RNA was extracted from *P*. *aeruginosa* PAO1 wild type, ∆4AQ or ∆5AQ grown to an OD_600_ 1.5 in the same conditions as described above for the microarray experiments. cDNA synthesis was performed from 1 μg of total purified RNA by using random hexamer primers and the iScript Reverse Transcription Supermix for RT-qPCR kit (BioRad). For RT-PCR, 50 ng of cDNA were PCR amplified with the GoTaq Polymerase (Promega) and primers FW*pqsE*RT and RV*pqsE*RT (for *pqsE*), FW*pqsE*-*phnA* and RV*pqsE*-*phnA* (for transcript spanning from *pqsE* to *phnA*), or FW*phnA*RT and RV*phnA*RT (for *phnA*) (**S3 Table**). After 5 min of denaturation at 95°C, the following reaction cycle was used for 30 cycles: 95°C for 30 s, 60°C for 30 s, and 72°C for 1 min. The PCR products were analysed on a 1% (w/v) agarose gel and stained with Midori Green DNA Stain (Nippon Genetis Europe GmbH).

Real-time PCRs were performed using the iTaq Universal SYBR Green Supermix (BioRad) and primers listed in **S3 Table**. Gene-specific primers employed in this analysis were designed using the Primer-Blast software (www.ncbi.nlm.nih.gov/tools/primer-blast) to avoid nonspecific amplification of *P*. *aeruginosa* DNA. The reaction procedure involved incubation at 95°C for 1 min and 40 cycles of amplification at 95°C for 10 s and 60°C for 45 s. Fluorescence was registered in the last 15 s of the 60°C step. 16S ribosomal RNA was chosen as the internal control to normalize the Real Time PCR data in each single run, and to calculate the relative fold change in gene expression by using the 2^-∆∆Ct^ method. The analysis was performed in duplicate on three technical replicates.

### Measurements of promoter activity, pyoverdine, AQ and anthranilate concentrations

Bioluminescence was determined as a function of cell density using an automated luminometer-spectrometer (GENios Pro), as previously described [27]. Pyoverdine was quantified as OD_405_ of culture supernatants appropriately diluted in 100 mM Tris-HCl (pH 8.0), and normalized for bacterial cell density (OD_600_) [53]. AQs were quantified by LC-MS/MS after extracting cultures with acidified ethyl acetate [54]. Anthranilate levels were determined using quantitative LC-MS/MS following extraction of bacterial cell pellets with 80% (v/v) methanol. MS analysis was conducted under positive electrospray conditions (+ES) with the MS in MRM (multiple reaction monitoring) mode. The precursor-product ion mass transition used for the MRM detection was *m/z* 138.1–120.1. The relevant chromatographic peaks were compared to those of an anthranilate standard at a range of known concentrations.

For all the assays, the average data and standard deviations were calculated from at least three independent experiments.

### FUR titration assay

The binding of Fur to the P*pqsA* and P*pqsR* promoter regions was investigated by transforming the miniCTX-P*pqsA*::*lux* [27] and miniCTX-P*pqsR*::*lux* plasmids into *E*. *coli* H1717 competent cells [49]. As positive and negative controls miniCTX-P*pchR*::*lux* and miniCTX-*lux* plasmids respectively were used. P*pchR*::*lux* was obtained by cloning a PCR fragment amplified from PAO1 with FWP*pchR* and RVP*pchR* (**S3 Table**). The resulting *E*. *coli* strains were grown for 16 h in LB broth supplemented with 10 μg ml^-1^ Tc at 37°C, washed twice with saline, and then isolated on MacConkey agar supplemented with 10 μg ml^-1^ Tc and 20 μM FeSO_4_, as previously described [51]. Colony colour was checked after 24 h of incubation at 37°C.

## Supporting Information

S1 Table Genes whose transcription is controlled by HHQ, PQS and/or PqsE. (PDF)

S2 Table Bacterial strains and plasmids. (PDF)

S3 Table Oligonucleotides. (PDF)

S1 Fig Validation of the *P*. *aeruginosa* ∆4AQ strain. (**A**) Levels of HHQ (white bars), PQS (light-grey bars) and HQNO (dark-grey bars) quantified by LC-MS/MS analysis in culture supernatants of *P*. *aeruginosa* ∆4AQ grown in LB or in LB supplemented with 40 μM HHQ, PQS, or HQNO, or with 1 mM IPTG, as indicated. AQ levels were quantified in supernatants from three independent cultures grown to OD_600_ 1.5. (**B**) Fold change in *pqsE* transcript levels measured by Real Time PCR in RNA extracted from ∆4AQ strains grown as in (**A**). Data were normalized to the *pqsE* RNA level in the wild type. (**C**) Growth curves of *P*. *aeruginosa* ∆4AQ grown in LB or in LB supplemented with 40 μM HHQ, PQS, or HQNO, or with 1 mM IPTG, as indicated.
(PDF)

S2 Fig The *pqsE* RNA transcript does not promote pyocyanin production. Pyocyanin production (**A**) and *pqsE* RNA levels measured by Real Time PCR (**B**) in *P*. *aeruginosa* ∆*pqsA* ∆*pqsE* double mutant strains carrying the pME6032 empty vector or pME6032-derivative plasmids for IPTG-inducible expression of wild type *pqsE* (pME-*pqsE*), or *pqsE* mutated variants lacking the first two codons (pME-*pqsE*∆1–6) or with a nucleotide insertion after the ATG to alter the protein frame (pME-*pqsE*NoFrame). Culture supernatants and total RNAs are from the indicated strains grown to an OD_600_ of 1.5 in LB supplemented with 1 mM IPTG. For the Real time PCR analysis, data are normalized to the *pqsE* RNA level measured in parallel in the *P*. *aeruginosa* PAO1 wild type.
(PDF)

S3 Fig RT-PCR analysis showing co-transcription of *pqsE* and *phnA*. Amplification of cDNAs retro-transcribed from RNA extracted from (**A**) *P*. *aeruginosa* PAO1 wild type grown in LB and (**B**) *P*. *aeruginosa* ∆4AQ grown in LB supplemented with 1 mM IPTG, to an OD_600_ of 1.5. A 200 bp DNA region within the *pqsE* gene (*pqsE*), a 280 bp DNA region spanning from 97 bp upstream of the *pqsE* stop codon to 68 bp downstream of the *phnA* start codon (*pqsE-phnA*), and a 200 bp DNA region inside the *phnA* gene (*phnA*) were amplified from: 1, PAO1 genomic DNA (positive control); 2, cDNA; 3, the corresponding RNA (negative control). L, GeneRuler 100 bp DNA Ladder Plus (MBI Fermentas).
(PDF)

S4 Fig PqsE does not affect anthranilate production and P*phzA2* activity, while it positively controls P*phzA1* activity. (**A**) Anthranilate was quantified by LC-MS/MS analysis in *P*. *aeruginosa* wild type and ∆4AQ grown to an OD_600_ of 1.5 in LB or in LB supplemented with 1 mM IPTG. Standard deviations are based on the mean values of three parallel cultures. (**B**) Maximal P*phzA1* and P*phzA2* promoter activity measured in *P*. *aeruginosa* PAO1 wild type and in the ∆*pqsAHLE* strain carrying the pUCP18 empty vector or the pUCP*pqsE* plasmid for constitutive expression of *pqsE*. Strains were grown in LB or in LB supplemented with 40 μM HHQ or PQS. Promoter activity is reported as Relative Light Units (RLU)/OD_600_.
(PDF)

S5 Fig AQs and PqsE do not affect P*pqsH* and P*pqsL* activity. Maximal promoter activity measured in *P*. *aeruginosa* ∆4AQ strains carrying the transcriptional fusions (**A**) P*pqsH*::*lux* or (**B**) P*pqsL*::*lux*. Strains were grown in LB or in LB supplemented with 40 μM AQs or 1 mM IPTG, as indicated below the graphs. Promoter activity is reported as Relative Light Units (RLU)/OD_600_.
(PDF)

S6 Fig The iron-chelators 2,2’-dipyridyl and deferiprone do not increase P*pqsA* activity. Maximal promoter activity in strains carrying the transcriptional fusions P*pqsA*::*lux* (**A** and **B**) or P*pqsR*::*lux* (**C** and **D**). Strains were grown in LB or in LB supplemented with 40 μM PQS, 500 μM 2,2’-dipyridyl (DIP), or 160 μM deferiprone (DEF), as indicated. Diamonds indicate the pyoverdine levels measured in parallel in culture supernatants. Promoter activity is reported as Relative Light Units (RLU)/OD_600_; pyoverdine levels are reported as OD_405_ normalized to cell density (OD_600_).
(PDF)

S7 Fig Impact of Fur and PvdS on the P*pqsA* and P*pqsR* promoter regions. (**A-D**) Maximal promoter activity in the strains carrying the transcriptional fusions P*pqsA*::*lux* (**A** and **B**) or P*pqsR*::*lux* (**C** and **D**). White bars indicate the *pvdS*-proficient genetic backgrounds (∆4AQ and ∆5AQ); grey bars indicate the *pvdS*-mutant genetic backgrounds (∆4AQ∆*pvdS* and ∆5AQ∆*pvdS*). Strains were grown in LB or in LB supplemented with 40 μM HHQ or PQS, as indicated. Diamonds indicate the pyoverdine levels measured in culture supernatants in the *pvdS*-proficient (white diamonds) or *pvdS*-mutant (grey diamonds) genetic backgrounds. Promoter activity is reported as Relative Light Units (RLU)/OD_600_; pyoverdine levels are reported as OD_405_ normalized to cell density (OD_600_). (**E**) Maximal P*pqsA*::*lux* and P*pqsR*::*lux* promoter activity in the wild type (white bars) and ∆*pvdS* (grey bars) strains grown in LB. Promoter activity is reported as Relative Light Units (RLU)/OD_600_. (**F**) *E*. *coli* H1717 cells containing the plasmids indicated and grown for 24 h at 37°C on McConkey agar supplemented with 10 μg ml^-1^ Tc and 20 μM FeSO_4_. Red-staining indicates the ability to ferment lactose and hence the binding of Fur to the target promoter. miniCTX-P*pchR*::*lux*, positive control (red colonies); miniCTX-*lux*, negative control (white colonies).
(PDF)
