# Supplementary material for: Unravelling the Genome-Wide Contributions of Specific 2-Alkyl-4-Quinolones and PqsE to Quorum Sensing in Pseudomonas aeruginosa
Source: PLoS Pathog. 2016 Nov 16;12(11):e1006029. doi: 10.1371/journal.ppat.1006029 (PMC5112799; doi:10.1371/journal.ppat.1006029)
Supplement: S1 Table — (PDF) [file ppat.1006029.s001.pdf]

Table S1. Genes whose transcription is controlled by HHQ, PQS and/or PqsE

PA number [a]	Gene name [a]	HHQ [b]	PQS [c]	PqsE [d]	Product name [a]
PA0051	*phzH*			3.2	Potential phenazine-modifying enzyme
PA0083	*tssB1*			-5.1	TssB1
PA0084	*tssC1*			-3.5	TssC1
PA0129	*bauD*		-5.5		Amino acid permease
PA0130	*bauC*		-5.5		3-oxopropanoate dehydrogenase
PA0131	*bauB*		-7.5		BauB
PA0132	*bauA*		-6.9		Beta-alanine:pyruvate transaminase
PA0187[J‡]				6.0	Hypothetical protein
PA0243[‡]				3.2	Probable transcriptional regulator
PA0263	*hcpC*		-4.3		Secreted protein Hcp
PA0269[J‡]				3.3	Conserved hypothetical protein
PA0270[‡]				2.8	Hypothetical protein
PA0271[J‡]				3.4	Hypothetical protein
PA0298	*spuB*			-2.8	Glutamylpolyamine synthetase
PA0299	*spuC*			-2.6	Polyamine:pyruvate transaminase
PA0303	*spuG*			-2.8	Polyamine transport protein PotH
PA0328	*aaaA*			-3.7	arginine-specific autotransporter, AaaA
PA0423	*pasP*		2.5		PasP
PA0431				2.7	Hypothetical protein
PA0432	*sahH*			2.9	S-adenosyl-L-homocysteine hydrolase
**PA0471**	***fiuR***		2.7		FiuR
**PA0472**	***fiuI***		2.5		FiuI
PA0480[J‡]				2.7	Probable hydrolase
PA0509	*nirN*		-16.0	-16.0	NirN
PA0510	*nirE*		-18.8	-13.1	NirE
PA0511	*nirJ*		-50.3	-22.1	Heme *d1* biosynthesis protein NirJ
PA0512	*nirH*		-22.4	-14.4	NirH
PA0513	*nirG*		-17.2	-13.5	NirG
PA0514	*nirL*		-27.2	-16.1	Heme *d1* biosynthesis protein NirL
PA0515	*nirD*		-75.3	-26.8	Probable transcriptional regulator
PA0516	*nirF*		-27.3	-12.8	Heme *d1* biosynthesis protein NirF
PA0517	*nirC*		-87.7	-21.5	Probable *c*-type cytochrome precursor
PA0518	*nirM*		-109.9	-28.6	Cytochrome *c551* precursor
PA0519	*nirS*		-101.2	-41.9	Nitrite reductase precursor
PA0520	*nirQ*		-4.7	-3.0	Regulatory protein NirQ
PA0521	*nirO*		-3.6	-3.2	Probable cytochrome *c* oxidase subunit
PA0523	*norC*		-32.4	-25.6	Nitric-oxide reductase subunit C
PA0524	*norB*		-41.0	-34.3	Nitric-oxide reductase subunit B
PA0525	*norD*		-7.2	-7.4	Probable denitrification protein NorD
PA0526			-20.1	-12.4	Hypothetical protein
PA0546	*metK*			3.0	Methionine adenosyltransferase
PA0746				-2.6	Probable acyl-CoA dehydrogenase

PA number [a]	Gene name [a]	HHQ [b]	PQS [c]	PqsE [d]	Product name [a]
PA0792	*prpD*		2.9		Propionate catabolic protein PrpD
PA0794			-3.7	-3.2	Probable aconitate hydratase
PA0795	*prpC*			-3.5	Citrate synthase 2
PA0796	*prpB*			-4.6	Carboxyphosphonoenolpyruvate phosphonomutase
PA0848[§]	*aphB*			2.5	alkyl hydroperoxide reductase, AhpB
PA0852	*cbpD*			4.1	Chitin-binding protein CbpD precursor
PA0918			-14.3		Cytochrome *b561*
**PA0931**	***pirA***		2.6		Ferric enterobactin receptor PirA
PA0997*[⌡‡◊]	*pqsB*	6.7	17.5		PqsB
PA0998*[⌡‡◊]	*pqsC*	5.5	16.1		PqsC
PA0999*[⌡‡◊]	*pqsD*	5.8	15.7		3-oxoacyl-[acyl-carrier-protein] synthase III
PA1000*[⌡◊]	*pqsE*			22.8	Quinolone signal response protein
PA1001*[⌡◊]	*phnA*			26.2	Anthranilate synthase component I
PA1002*[⌡◊]	*phnB*			22.4	Anthranilate synthase component II
PA1070	*braG*			-3.5	Branched-chain amino acid transport protein BraG
PA1071	*braF*			-3.1	Branched-chain amino acid transport protein BraF
PA1072	*braE*			-4.0	Branched-chain amino acid transport protein BraE
PA1073	*braD*			-5.3	Branched-chain amino acid transport protein BraD
**PA1134**			3.3		Hypothetical protein
PA1172	*napC*		-5.7		Cytochrome *c*-type protein NapC
PA1173	*napB*		-8.9		Cytochrome *c*-type protein NapB precursor
PA1174[§]	*napA*		-6.5		Periplasmic nitrate reductase protein NapA
PA1195			-3.4		Hypothetical protein
PA1212[‡]				2.8	Probable MFS transporter
PA1213[⌡]				3.7	Hypothetical protein
PA1214				3.6	Hypothetical protein
PA1215				4.1	Hypothetical protein
PA1216*				4.9	Hypothetical protein
PA1217				5.8	Probable 2-isopropylmalate synthase
PA1218[⌡]				5.5	Hypothetical protein
**PA1245**[§⌡]	***aprX***		3.9		AprX
**PA1300**			11.1		Probable sigma-70 factor, ECF subfamily
**PA1301**[‡]			6.1		Probable transmembrane sensor
**PA1318**[⌡]	***cyoB***		2.9		Cytochrome *o*-ubiquinol oxidase subunit I
PA1551	*fixG*		-3.7		Probable ferredoxin
PA1562	*acnA*		-2.6		Aconitate hydratase 1
PA1565	*pauB2*		-9.7	-3.8	FAD-dependent oxidoreductase
PA1583	*sdhA*		-2.6		Succinate dehydrogenase (flavoprotein subunit)
PA1602			-2.7		Probable oxidoreductase
PA1659	*hsiF2*		-2.5		HsiF2
PA1706	*pcrV*		2.7		Type III secretion protein PcrV
PA1707	*pcrH*		3.1		Regulatory protein PcrH
PA1708	*popB*		5.6		Translocator protein PopB
PA1709	*popD*		3.0		Translocator outer membrane protein PopD precursor

PA number [a]	Gene name [a]	HHQ [b]	PQS [c]	PqsE [d]	Product name [a]
PA1710	*exsC*		3.5		ExsC exoenzyme S synthesis protein C precursor
PA1711	*exsE*		3.1		ExsE
PA1712	*exsB*		2.6		Exoenzyme S synthesis protein B
PA1718	*pscE*		4.3		Type III export protein PscE
PA1869				2.8	Probable acyl carrier protein
PA1888				2.8	Hypothetical protein
PA1897			-3.1		Hypothetical protein
PA1901[J]	*phzC2*			5.5	Phenazine biosynthesis protein PhzC
PA1902[§]	*phzD2*			7.5	Phenazine biosynthesis protein PhzD
PA1903[J]	*phzE2*			8.8	Phenazine biosynthesis protein PhzE
PA1904	*phzF2*			10.3	Probable phenazine biosynthesis protein
PA1905	*phzG2*			9.7	Probable pyridoxamine 5'-phosphate oxidase
PA1912	*femI*		3.6		ECF sigma factor, FemI
PA1946	*rbsB*			-2.6	Binding protein component precursor of ABC ribose transporter
PA2014	*liuB*			-2.6	Methylcrotonyl-CoA carboxylase, beta-subunit
PA2030				3.7	Hypothetical protein
PA2031*				3.7	Hypothetical protein
**PA2033**[J]			17.6		Hypothetical protein
**PA2034**			5.5		Hypothetical protein
PA2041				-4.7	Amino acid permease
PA2066[J‡]				3.0	Hypothetical protein
PA2068[J]				2.7	Probable MFS transporter
PA2069*[J]				3.9	Probable carbamoyl transferase
PA2193*	*hcnA*			3.6	Hydrogen cyanide synthase HcnA
PA2194*	*hcnB*			3.1	Hydrogen cyanide synthase HcnB
PA2195*	*hcnC*			3.0	Hydrogen cyanide synthase HcnC
PA2264			-3.4		Conserved hypothetical protein
PA2265			-3.7		Gluconate dehydrogenase
PA2274*[J]				5.4	Hypothetical protein
PA2300*[J◊]	*chiC*			18.7	Chitinase
**PA2383**			4.5		Probable transcriptional regulator
**PA2384**[§]			42.2		Hypothetical protein
**PA2385**	***pvdQ***		29.1		3-oxo-C_{12}-homoserine lactone acylase PvdQ
**PA2386**	***pvdA***		113.8		L-ornithine N5-oxygenase
**PA2389**	***pvdR***		7.3		PvdR
**PA2390**	***pvdT***		4.1		PvdT
**PA2391**	***opmQ***		3.3		Probable outer membrane protein precursor
**PA2392**	***pvdP***		7.9		PvdP
**PA2393**			55.0		Probable dipeptidase precursor
**PA2394**	***pvdN***		45.6		PvdN
**PA2395**	***pvdO***		27.3		PvdO
**PA2396**	***pvdF***		49.0		Pyoverdine synthetase F
**PA2397**	***pvdE***		48.2		Pyoverdine biosynthesis protein PvdE
**PA2398**	***fpvA***		16.4		Ferripyoverdine receptor

PA number [a]	Gene name [a]	HHQ [b]	PQS [c]	PqsE [d]	Product name [a]
**PA2399**	***pvdD***		18.2		Pyoverdine synthetase D
**PA2400**	***pvdJ***		21.9		PvdJ
**PA2402**			23.1		Probable non-ribosomal peptide synthetase
**PA2403**			4.7		Hypothetical protein
**PA2404**			3.6		Hypothetical protein
**PA2405**[§]			3.9		Hypothetical protein
**PA2406**			3.4		Hypothetical protein
**PA2407**			4.6		Probable adhesion protein
**PA2408**			3.7		Probable ATP-binding component of ABC transporter
**PA2409**			3.5		Probable permease of ABC transporter
**PA2410**			3.0		Hypothetical protein
**PA2411**			30.2		Probable thioesterase
**PA2412**[§J]			70.7		Conserved hypothetical protein
**PA2413**[J]	***pvdH***		43.1		L-2,4-diaminobutyrate:2-ketoglutarate 4-aminotransferase
**PA2424**	***pvdL***		26.2		PvdL
**PA2425**	***pvdG***		11.6		PvdG
**PA2426**	***pvdS***		43.9		Sigma factor PvdS
**PA2427**			21.3		Hypothetical protein
PA2444	*glyA2*			-3.9	Serine hydroxymethyltransferase
PA2448[‡]				3.7	Hypothetical protein
**PA2451**			5.1		Hypothetical protein
**PA2452**			48.2		Hypothetical protein
PA2481			-2.9		Hypothetical protein
PA2482			-2.7		Probable cytochrome *c*
PA2509[J‡]	*catB*			4.3	Muconate cycloisomerase I
PA2511[‡]	*antR*			3.2	AntR
**PA2531**			3.2		Probable aminotransferase
PA2554				-2.7	Probable short-chain dehydrogenase
PA2555				-2.8	Probable AMP-binding enzyme
PA2557				-3.3	Probable AMP-binding enzyme
PA2564	*tam*			4.6	Hypothetical protein
PA2565				3.5	Hypothetical protein
PA2570*[J◊]	*lecA*			26.3	LecA lectin
PA2588				2.7	Probable transcriptional regulator
PA2682				3.0	Conserved hypothetical protein
PA2765			-2.9		Hypothetical protein
PA2862	*lipA*			-8.7	Lactonizing lipase precursor
PA2953			-2.9		Electron transfer flavoprotein-ubiquinone oxidoreductase
PA3032[§]	*snr1*			5.4	Cytochrome *c* Snr1
PA3120	*leuD*		-2.5		3-isopropylmalate dehydratase small subunit
PA3121	*leuC*		-3.6		3-isopropylmalate dehydratase large subunit
PA3190	*gltB*			-3.3	Probable binding protein component of ABC sugar transporter
PA3192	*gltR*			-2.6	Two-component response regulator GltR
PA3195*	*gapA*			-4.6	Glyceraldehyde 3-phosphate dehydrogenase

PA number [a]	Gene name [a]	HHQ [b]	PQS [c]	PqsE [d]	Product name [a]
PA3300	*fadD2*			-2.5	Long-chain-fatty-acid-CoA ligase
PA3361*[◊]	*lecB*			8.5	Fucose-binding lectin LecB
PA3369*				3.1	Hypothetical protein
PA3391	*nosR*		-4.6	-4.1	Regulatory protein NosR
PA3392*	*nosZ*		-72.7	-58.8	Nitrous-oxide reductase precursor
PA3393	*nosD*		-8.8	-9.0	NosD protein
PA3394	*nosF*		-6.3	-5.8	NosF protein
PA3395	*nosY*		-3.9	-3.2	NosY protein
PA3396	*nosL*		-3.4	-2.7	NosL protein
**PA3407**	***hasAp***		76.6		Heme acquisition protein HasAp
**PA3408**	***hasR***		6.4		Heme uptake outer membrane receptor HasR precursor
**PA3410**	***hasI***		7.6		HasI
PA3441	*ssuF*		10.2		Probable molybdopterin-binding protein
PA3444	*ssuD*		4.2		Conserved hypothetical protein
PA3452	*mqoA*		2.6		Malate:quinone oxidoreductase
PA3478*	*rhlB*			3.6	Rhamnosyltransferase chain B
PA3479	*rhlA*			3.6	Rhamnosyltransferase chain A
PA3484	*tse3*			-2.6	Tse3
PA3520*[§]				4.0	Hypothetical protein
PA3524	*gloA1*		-2.7		Lactoylglutathione lyase
**PA3530**	***bfd***		18.6		Bacterioferritin-associated ferredoxin Bfd
PA3568	*ymmS*			-8.9	Probable acetyl-CoA synthetase
PA3569	*mmsB*			-5.3	3-hydroxyisobutyrate dehydrogenase
PA3570	*mmsA*			-3.9	Methylmalonate-semialdehyde dehydrogenase
PA3600	*rpl36*		3.0		Conserved hypothetical protein
PA3601	*ykgM*		2.7		Conserved hypothetical protein
PA3602	*yerD*		-6.4		Conserved hypothetical protein
PA3709				-6.4	Probable MFS transporter
PA3710				-5.0	Probable GMC-type oxidoreductase
PA3734				4.8	Hypothetical protein
PA3784			-6.0		Hypothetical protein
PA3785			-7.0		Conserved hypothetical protein
PA3790	*oprC*		-3.7		Putative copper transport outer membrane porin OprC
PA3841	*exoS*		2.6		Exoenzyme S
PA3842	*spcS*		3.9		Specific *Pseudomonas* chaperone for ExoS, SpcS
PA3866			2.6		Pyocin protein
PA3870	*moaA1*		-2.7		Molybdopterin biosynthetic protein A1
PA3872	*narI*		-7.0	-5.0	Respiratory nitrate reductase gamma chain
PA3873	*narJ*		-3.6	-3.1	Respiratory nitrate reductase delta chain
PA3874	*narH*		-6.5	-3.8	Respiratory nitrate reductase beta chain
**PA3899**	*fecI*		3.4		FecI
PA3938	*tauA*		2.7		Probable periplasmic taurine-binding protein precursor
PA4023	*eutP*			-4.5	Probable transport protein
PA4024	*eutB*			-2.8	Ethanolamine ammonia-lyase large subunit

PA number [a]	Gene name [a]	HHQ [b]	PQS [c]	PqsE [d]	Product name [a]
PA4078*				12.1	Probable nonribosomal peptide synthetase
PA4131[§]			-5.8		Probable iron-sulfur protein
PA4132			-3.1		Conserved hypothetical protein
PA4133	*ccoN*		-5.2		Cytochrome c oxidase subunit (cbb3-type)
PA4134[‡]			-6.1		Hypothetical protein
PA4140[‡]			5.3		Hypothetical protein
PA4141*[§‡]			4.4	5.2	Hypothetical protein
PA4142[‡]			4.3	4.8	Probable secretion protein
**PA4168**	***fpvB***		4.0		Second ferric pyoverdine receptor FpvB
**PA4175**	***prpL***		3.7		PrpL, protease IV
PA4205*[J◊]	*mexG*			25.0	Hypothetical protein
PA4206*[J◊]	*mexH*			16.4	Probable RND efflux membrane fusion protein precursor
PA4207*[J◊]	*mexI*			18.5	Probable RND efflux transporter
PA4208*[J◊]	*opmD*			11.6	Probable outer membrane protein precursor
PA4209*[J◊]	*phzM*			4.1	Probable phenazine-specific methyltransferase
PA4210[◊]	*phzA*			10.2	Probable phenazine biosynthesis protein
PA4211*[◊]	*phzB*			5.6	Probable phenazine biosynthesis protein
PA4217*[§◊]	*phzS*			9.0	Flavin-containing monooxygenase
**PA4218**[§J]	***ampP***		22.6		AmpP
**PA4219**	***yfpB***		10.7		AmpO
**PA4220**[§]	***fptB***		30.3		Hypothetical protein
**PA4221**[§J‡]	***fptA***		23.6		Fe(III)-pyochelin outer membrane receptor precursor
**PA4222**[§J‡]	***pchI***		13.8		Probable ATP-binding component of ABC transporter
**PA4223**[§]	***pchH***		13.7		Probable ATP-binding component of ABC transporter
**PA4224**[§]	***pchG***		40.1		Pyochelin biosynthetic protein PchG
**PA4225**[§J‡]	***pchF***		37.0		Pyochelin synthetase
**PA4226**[§J‡]	***pchE***		28.0		Dihydroaeruginoic acid synthetase
**PA4227**[J‡]	***pchR***		11.2		Transcriptional regulator PchR
**PA4228**[§J‡]	***pchD***		34.1		Pyochelin biosynthesis protein PchD
**PA4229**[§J]	***pchC***		31.5		Pyochelin biosynthetic protein PchC
**PA4230**[§J]	***pchB***		47.3		Salicylate biosynthesis protein PchB
**PA4231**[§J‡]	***pchA***		44.0		Salicylate biosynthesis isochorismate synthase
PA4236[§]	*katA*		-5.5		Catalase
PA4333	*fumA*		-3.4		Probable fumarase
PA4366	*sodB*		-3.8		Superoxide dismutase
**PA4370**	***icmP***		2.6		Insulin-cleaving metalloproteinase outer membrane protein
PA4384				3.0	Hypothetical protein
PA4429			-2.9		Probable cytochrome *c1* precursor
PA4430			-3.1		Probable cytochrome *b*
PA4431			-2.8		Probable iron-sulfur protein
**PA4467**			12.9		Hypothetical protein
**PA4468**[§]	***sodA***		89.4		Superoxide dismutase
**PA4469**[§J]	***orfX***		136.5		OrfX
**PA4470**[§J]	***fumC1***		114.7		Fumarate hydratase

PA number [a]	Gene name [a]	HHQ [b]	PQS [c]	PqsE [d]	Product name [a]
**PA4471**[§∫]	***fagA***		58.6		FagA
PA4500				-2.6	Probable binding protein component of ABC transporter
PA4501	*opdD*			-5.4	Glycine-glutamate dipeptide porin OpdP
PA4502				-2.8	Probable binding protein component of ABC transporter
PA4519	*speC*			2.7	Ornithine decarboxylase
**PA4570**			57.4		Hypothetical protein
PA4587*[§]	*ccpR*		-12.3		Cytochrome *c551* peroxidase precursor
PA4613[§‡]	*katB*			2.7	Catalase
PA4623				2.5	Hypothetical protein
PA4648	*cupE1*			3.0	Pilin subunit CupE1
**PA4708**	***phuT***		4.2		Heme-transport protein, PhuT
**PA4709**	***phuS***		4.5		PhuS
**PA4710**	***phuR***		9.2		Heme/Haemoglobin uptake outer membrane receptor PhuR
PA4774				-2.5	Hypothetical protein
PA4810	*fdnI*		-3.0		Nitrate-inducible formate dehydrogenase, gamma subunit
PA4811[§]	*fdnH*		-4.5		Nitrate-inducible formate dehydrogenase, beta subunit
PA4812[§]	*fdnG*		-5.3		Formate dehydrogenase-O, major subunit
PA4880[§]			-10.4		Probable bacterioferritin
**PA4896**			3.5		Probable sigma-70 factor, ECF subfamily
PA5058	*phaC2*			2.5	Poly(3-hydroxyalkanoic acid) synthase 2
PA5082	*dguC*			-13.2	DguC
PA5098	*hutH*			-3.5	Histidine ammonia-lyase
PA5100	*hutU*			-3.2	Urocanase
PA5153				-2.6	Amino acid ABC transporter periplasmic binding protein
PA5154				-4.5	Probable permease of ABC transporter
PA5167	*dctP*			-3.4	DctP
PA5168	*dctQ*			-5.6	DctQ
PA5169	*dctM*			-7.0	DctM
PA5300	*cycB*		-3.4		Cytochrome *c5*
PA5355	*glcD*		-3.5		Glycolate oxidase subunit GlcD
PA5380	*gbdR*		-2.6		GbdR
PA5396			-4.3		Hypothetical protein
PA5397			-4.6		Hypothetical protein
PA5410	*gbcA*		-5.7		GbcA
PA5460[§]				3.7	Hypothetical protein

[a] PA number, gene name and product name are from the *Pseudomonas* Genome Database [13]. Genes previously reported controlled by iron-starvation are in bold characters [40,41]; genes controlled by PQS *via* a PqsR-independent and iron starvation-independent signalling pathway(s) are underlined. *, genes whose transcription was altered in the Δ*pqsR* mutant with respect to the wild type strain [10]; §, genes whose transcription was altered upon exogenous PQS provision [28]; ∫, genes whose transcription was altered in the Δ*pqsA* mutant with respect to the wild type strain [11]; ‡, genes whose transcription was altered upon PqsE overexpression [11]; ◊, genes whose transcription was altered in the Δ*pqsH* mutant with respect to the wild type strain [23]. RND, Resistance-Nodulation-Cell division; MFS, major facilitator superfamily.

[b] Fold change in gene expression in *P. aeruginosa* PAO1 Δ4AQ grown in LB supplemented with 40 µM HHQ with respect to the same strain grown in LB.

[c] Fold change in gene expression in *P. aeruginosa* PAO1 Δ4AQ grown in LB supplemented with 40 µM PQS with respect to the same strain grown in LB.

[d] Fold change in gene expression in *P. aeruginosa* PAO1 Δ4AQ grown in LB supplemented with 1 mM IPTG (to induce PqsE expression) with respect to the same strain grown in LB.
