# Supplementary material for: Unravelling the Genome-Wide Contributions of Specific 2-Alkyl-4-Quinolones and PqsE to Quorum Sensing in Pseudomonas aeruginosa
Source: PLoS Pathog. 2016 Nov 16;12(11):e1006029. doi: 10.1371/journal.ppat.1006029 (PMC5112799; doi:10.1371/journal.ppat.1006029)
Supplement: S2 Table — (PDF) [file ppat.1006029.s002.pdf]

Table S2. Bacterial strains and plasmids

Strains/Plasmids	Relevant characteristics and plasmids construction	Reference/Source
E. coli		
DH5α	Cloning strain.	[55]
S17.1λ*pir*	Conjugative strain for suicide plasmids.	[56]
H1717	Mutant strain carrying the P*fhuF*::*lacZ* transcriptional fusion that does not produce the siderophore enterochelin. Used for the Fur titration assay (FurTA).	[51]
P. aeruginosa		
PAO1	Nottingham collection wild type strain.	
Δ*pqsA* Δ*pqsH*	*pqsA* and *pqsH* double mutant of strain PAO1.	[27]
Δ*pqsA* Δ*pqsE*	*pqsA* and *pqsE* double mutant of strain PAO1.	[11]
Δ*pqsR*	*pqsR* mutant of strain PAO1.	This study
Δ4AQ	Quadruple mutant of PAO1 carrying in frame clear deletions of the *pqsA*, *pqsH*, and *pqsL* genes, in which *pqsE* is under the control of an IPTG-inducible promoter.	This study
Δ5AQ	Quintuple mutant of PAO1 carrying in frame clear deletions of the *pqsA*, *pqsH*, *pqsL*, and *pqsR* genes, in which *pqsE* is under the control of an IPTG-inducible promoter.	This study
Δ*pvdS*	*pvdS* mutant of strain PAO1.	This study
Δ4AQΔ*pvdS*	Δ4AQ strain carrying a deletion of the *pvdS* gene.	This study
Δ5AQΔ*pvdS*	Δ5AQ strain carrying a deletion of the *pvdS* gene.	This study
Δ*pqsAHLE*	Quadruple mutant of PAO1 carrying in frame clear deletions of the *pqsA*, *pqsH*, *pqsL* and *pqsE* genes.	This study
**Plasmids**		
pBluescript II KS(+)	Cloning vector; ColE1 replicon; Ap^R.	Stratagene
pDM4	Suicide vector; *sacBR*; *oriR6K*; Cm^R.	[57]
pME6032	pVS1-p15A shuttle expression (IPTG-inducible) vector, Tc^R.	[58]
miniCTX-*lux*	Promoter-probe vector containing the *luxCDABE* operon; Tc^R.	[59]
pEXΔ*pvdS*	pEX18 derivative used to introduce the *pvdS* mutation in PAO1 wild type, Δ4AQ and Δ5AQ.	[53]
pDM4*pqsE*ind	pDM4 derivative for the generation of the *pqsE*-inducible strain Δ4AQ.	[11]

Strains/Plasmids	Relevant characteristics and plasmids construction	Reference/Source
pDM4Δ*pqsE*	pDM4 derivative for the generation of the PAO1 Δ*pqsAHLE* mutant strain.	[11]
pDM4Δ*pqsR*	pDM4 derivative used to introduce the *pqsR* mutation in PAO1 wild type and in the Δ5AQ strain.	[24]
miniCTX-P*pqsA*::*lux*	miniCTX-*lux* derivative used to insert the P*pqsA*::*lux* fusion in the chromosome of different *P. aeruginosa* strains.	[27]
pUCP18	pUC18 derivative containing a stabilising fragment for maintenance in *Pseudomonas*; Ap^R, *E. coli* / Cb^R, *P. aeruginosa*.	[60]
pUCP*pqsE*	pUCP18 derivative for *pqsE* complementation; Ap^R.	[11]
pBS*pqsL*UP	The DNA fragnment encompassing the upstream region of the *pqsL* gene originated with primers FW*pqsL*UP and RV*pqsL*UP (Table S3) was cloned in pBluescript II KS(+) by XhoI-EcoRI restriction.	This study
pBS*pqsL*DOWN	The DNA fragment encompassing the downstream region of the *pqsL* gene originated with primers FW*pqsL*DOWN and RV*pqsL*DOWN (Table S3) was cloned in pBluescript II KS(+) by EcoRI-XbaI restriction.	This study
pDM4Δ*pqsL*	pDM4-derived plasmid used to introduce the *pqsL* mutation in *P. aeruginosa* Δ*pqsA* Δ*pqsH*. It contains the DNA fragments encompassing the upstream region *pqsL* (extracted by XhoI-EcoRI restriction from pBS*pqsL*UP) and the downstream region of *pqsL* (extracted by EcoRI-XbaI restriction from pBS*pqsL*DOWN), and cloned in pDM4 by XhoI-XbaI.	This study
miniCTX-P*pqsH*::*lux*	miniCTX-*lux* derivative used to insert the P*pqsH*::*lux* fusion in the chromosome of different *P. aeruginosa* strains. Obtained by cloning in miniCTX-*lux* with restriction enzymes XhoI-PstI a DNA region encompassing the P*pqsH* promoter amplified with primers FWP*pqsH* and RVP*pqsH* (Table S3).	This study
miniCTX-P*pqsL*::*lux*	miniCTX-*lux* derivative used to insert the P*pqsL*::*lux* fusion in the chromosome of different *P. aeruginosa* strains. Obtained by cloning in miniCTX-*lux* with restriction enzymes XhoI-PstI a DNA region encompassing the P*pqsL* promoter amplified with primers FWP*pqsL* and RVP*pqsL* (Table S3).	This study

Strains/Plasmids	Relevant characteristics and plasmids construction	Reference/Source
miniCTX-P*pqsR*::*lux*	miniCTX-*lux* derivative used to insert the P*pqsR*::*lux* fusion in the chromosome of different *P. aeruginosa* strains. Obtained by cloning in miniCTX-*lux* with restriction enzymes HindIII-EcoRI a DNA region encompassing the P*pqsR* promoter amplified with primers FWP*pqsR* and RVP*pqsR* (Table S3).	This study
miniCTX-P*pchR*::*lux*	miniCTX-*lux* derivative used to test the ability of Fur to bind the P*pchR* promoter (FurTA; posistive control). Obtained by cloning in miniCTX-*lux* with restriction enzymes XhoI-HindIII a DNA region encompassing the P*pchR* promoter amplified with primers FWP*pchR* and RVP*pchR* (Table S3).	This study
miniCTX-P*phzA1*::*lux*	miniCTX-*lux* derivative used to insert the P*phzA1*::*lux* fusion into the chromosome of *P. aeruginosa* Δ*pqsAHLE*. Obtained by cloning in miniCTX-*lux* with restriction enzymes HindIII-BamHI a DNA region encompassing the P*phzA1* promoter amplified with primers FWP*phzA1* and RVP*phzA1* (Table S3).	This study
miniCTX-P*phzA2*::*lux*	miniCTX-*lux* derivative used to insert the P*phzA2*::*lux* fusion into the chromosome of *P. aeruginosa* Δ*pqsAHLE*. Obtained by cloning in miniCTX-*lux* with restriction enzymes EcoRI-PstI a DNA region encompassing the P*phzA2* promoter amplified with primers FWP*phzA2* and RVP*phzA2* (Table S3).	This study
pME-*pqsE*	pME6032 derivative for the IPTG-dependent expression on *pqsE*. Obtained by cloning in pME6032 with restriction enzymes EcoRI-SacI a DNA region encompassing the *pqsE* gene amplified with primers FW*pqsE* and RV*pqsE* (Table S3).	This study
pME-*pqsE*Δ1-6	pME6032 derivative for the IPTG-dependent expression of a mutated variant of *pqsE* lacking the first two codons (ATGTTG). Obtained by cloning in pME6032 with restriction enzymes EcoRI-SacI a DNA region encompassing the *pqsE* gene amplified with primers FW*pqsE*Δ1-6 and RV*pqsE*Δ1-6 (Table S3).	This study

Strains/Plasmids	Relevant characteristics and plasmids construction	Reference/Source
pME-*pqsE*NoFrame	pME6032 derivative for the IPTG-dependent expression of a mutated variant of *pqsE* out of frame. Obtained by cloning in pME6032 with restriction enzymes EcoRI-SacI a DNA region encompassing the *pqsE* gene amplified with primers FW*pqsE*NoFrame and RV*pqsE* (Table S3).	This study

Additional references for Table S2:

55. Grant SG, Jessee J, Bloom FR, Hanahan D. Differential plasmid rescue from transgenic mouse DNAs into *Escherichia coli* methylation-restriction mutants. Proc Natl Acad Sci USA. 1990;87:4645-9.
56. Simon R, Priefer U, Puhler A. A broad host range mobilization system for *in vivo* genetic-engineering: transposon mutagenesis in Gram-negative bacteria. Biotechnology. 1983;1:784-91.
57. Milton DL, O'Toole R, Horstedt P, Wolf-Watz H. Flagellin A is essential for the virulence of *Vibrio anguillarum*. J Bacteriol. 1996;178:1310-9.
58. Heeb S, Blumer C, Haas D. Regulatory RNA as mediator in GacA/RsmA-dependent global control of exoproduct formation in *Pseudomonas fluorescens* CHA0. J Bacteriol. 2002;184:1046-56.
59. Becher A, Schweizer HP. Integration-proficient *Pseudomonas aeruginosa* vectors for isolation of single-copy chromosomal *lacZ* and *lux* gene fusions. Biotechniques. 2000;29:948-50.
60. Schweizer HP. *Escherichia-Pseudomonas* shuttle vectors derived from pUC18/19. Gene. 1991;97:109-21.
