# Supplementary material for: Unravelling the Genome-Wide Contributions of Specific 2-Alkyl-4-Quinolones and PqsE to Quorum Sensing in Pseudomonas aeruginosa
Source: PLoS Pathog. 2016 Nov 16;12(11):e1006029. doi: 10.1371/journal.ppat.1006029 (PMC5112799; doi:10.1371/journal.ppat.1006029)
Supplement: S3 Table — (PDF) [file ppat.1006029.s003.pdf]

Table S3. Oligonucleotides

Name	Sequence (5'-3') [a]	Restriction site [b]
FWP*pqsH*	CCGCTCGAGGGTCGTGCATGCTTGCCG	XhoI
RVP*pqsH*	TAACTGCAGTTGCTCCTTAGCAGCGGC	PstI
FWP*pqsL*	TCCGCTCGAGGATCGTCACCGTCAACTG	XhoI
RVP*pqsL*	TAACTGCAGCGTCATGGATGAGTCTCCG	PstI
FWP*pqsR*	TATAAGCTTAGGCCCTTGGTATTAACG	HindIII
RVP*pqsR*	TATGAATTCCCCTTATTCCTTTTATTG	EcoRI
FWP*pchR*	TATCTCGAGCGATCTCCGTGGATGCG	XhoI
RVP*pchR*	TATAAGCTTGGTCATCAGGTTTTCCTGTA	HindIII
FWP*phzA1*	GGCGGCAAGCTTCTCGTCGCTGTCGAT	HindIII
RVP*phzA1*	TTTCCCGGATCCGCTGACCGTTCATGC	BamHI
FWP*phzA2*	AGCGCAGAATTCCCACCGGCAGCGTTT	EcoRI
RVP*phzA2*	ACGCAACTGCAGGTTGTCGGTAAACCC	PstI
FW*pqsL*UP	CCGCTCGAGACCGTGGTCGATGGCGT	XhoI
RV*pqsL*UP	CCGGAATTCCGTCATGGATGAGTCTCC	EcoRI
FW*pqsL*DOWN	CCGGAATTCCTCGGCTGAACCCGCC	EcoRI
RV*pqsL*DOWN	TGCTCTAGACCTGTTCAATTACCCGAGCC	XbaI
FW*pqsB*	CCGCTCGAGCGACCAGGGCTATCGCA	XhoI
FW*pqsE*	TATGAATTCATGTTGAGGCTTTCGGCTCC	EcoRI
RV*pqsE*	TATGAGCTCTCAGTCCAGAGGCAGCGC	SacI
FW*pqsE*Δ1-6	TATGAATTCAGGCTTTCGGCTCC	EcoRI
RV*pqsE*Δ1-6	TATGAGCTCTGTCCCGTCTCAGTCCAG	SacI
FW*pqsE*NoFrame	TATGAATTCATGGTTGAGGCTTTCGGCTC	EcoRI
RV*pqsB*	CCGGAATTCCTTATGCATGAGCTTCTCC	EcoRI
FW16SRT	AGTACGGCCGCAAGGTTAAA	-
RV16SRT	CCCAACATCTCACGACACGA	-
FW*pchR*RT	CTCAGCGCACAGTTCCTTTC	-
RV*pchR*RT	CGAACACCTTGCGAAAGCC	-
FW*pvdS*RT	GGAACAACTGTCTACCCGCA	-
RV*pvdS*RT	GTAGCTGAGCTGTGCCTTGA	-
FW*lecA*RT	CAGGGCAGGTAACGTCGATT	-
RV*lecA*RT	CAACCCGGTATTGACCGGAA	-
FW*rhlA*RT	CATCTGCTCAACGACGACCGT	-
RV*rhlA*RT	TGCCGTTGATGAAATGCACG	-
FW*mexG*RT	CTGGCGAAGCTGTTCGACTA	-
RV*mexG*RT	TTGCTCCAGAAGGTGTGGAC	-
FW*nosR*RT	AGCTATCGGTGGTCAACGTG	-
RV*nosR*RT	GCGAGTTCGTTGAGCAGTTC	-

**Name**	**Sequence (5'-3')** [a]	**Restriction site** [b]
FW*hcpC*RT	TATCAGGAAGGCCACGAGGA	-
RV*hcpC*RT	CCACTGGATCTCGACCTTGG	-
FW*aprX*RT	CTGCCGATCAACGTCTCCTT	-
RV*aprX*RT	TACCGTAGAACTTGGCGCTG	-
FW*sodA*RT	GCTGACCATGGAGATCCACC	-
RV*sodA*RT	CGACATCACGGTCCAGAACA	-
FW*pqsE*RT	CGGTGTTCCTGCTGCGTC	-
RV*pqsE*RT	GACGCCAGGACCTGTACG	-
FW*phnA*RT	GCGACATGCTCAAGGTCGAT	-
RV*phnA*RT	CTGCCGCGATAGCCATCTT	-
FW*pqsE-phnA*	GTGGGCAGAGCGTCGACT	-
RV*pqsE-phnA*	GTGCGGTACTCCAGACTTTC	-

[a] Engineered restriction sites are underlined.

[b] -, no restriction site introduced.
