# Supplementary material for: Unravelling the Genome-Wide Contributions of Specific 2-Alkyl-4-Quinolones and PqsE to Quorum Sensing in Pseudomonas aeruginosa
Source: PLoS Pathog. 2016 Nov 16;12(11):e1006029. doi: 10.1371/journal.ppat.1006029 (PMC5112799; doi:10.1371/journal.ppat.1006029)
Supplement: S1 Fig — (A) Levels of HHQ (white bars), PQS (light-grey bars) and HQNO (dark-grey bars) quantified by LC-MS/MS analysis in culture supernatants of P. aeruginosa ∆4AQ grown in LB or in LB supplemented with 40 μM HHQ, PQS, or HQNO, or with 1 mM IPTG, as indicated. AQ levels were quantified in supernatants from three independent cultures grown to OD600 1.5. (B) Fold change in pqsE transcript levels measured by Real Time PCR in RNA extracted from ∆4AQ strains grown as in (A). Data were normalized to the pqsE RNA level in the wild type. (C) Growth curves of P. aeruginosa ∆4AQ grown in LB or in LB supplemented with 40 μM HHQ, PQS, or HQNO, or with 1 mM IPTG, as indicated. (PDF) [file ppat.1006029.s004.pdf]

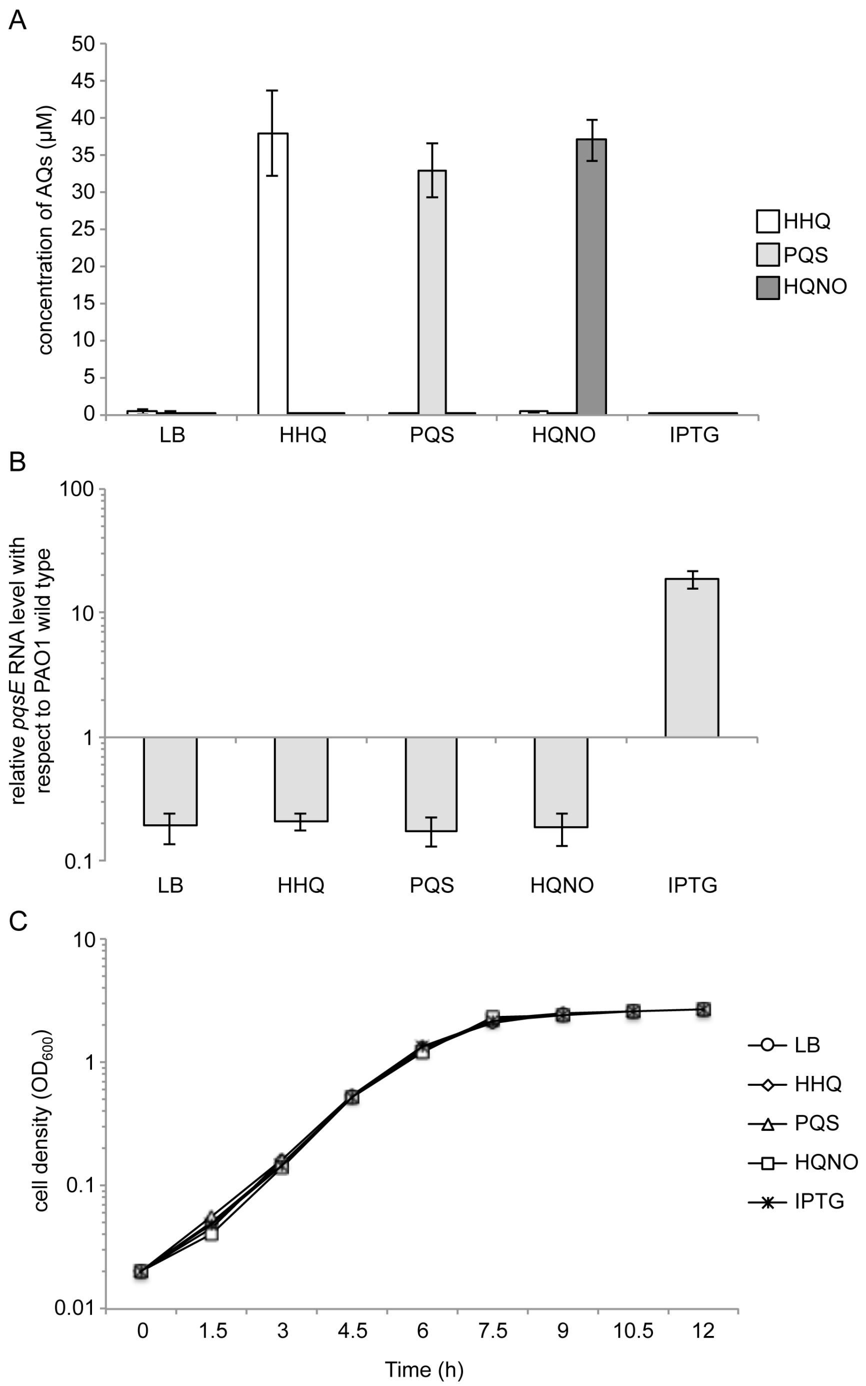

Fig S1. Validation of the *P. aeruginosa* Δ4AQ strain

(**A**) Levels of HHQ (white bars), PQS (light-grey bars) and HQNO (dark-grey bars) quantified by LC-MS/MS analysis in culture supernatants of *P. aeruginosa* Δ4AQ grown in LB or in LB supplemented with 40 µM HHQ, PQS, or HQNO, or with 1 mM IPTG, as indicated. AQ levels were quantified in supernatants from three independent cultures grown to OD_{600} 1.5. (**B**) Fold change in *pqsE* transcript levels measured by Real Time PCR in RNA extracted from Δ4AQ strains grown as in (**A**). Data were normalized to the *pqsE* RNA level in the wild type. (**C**) Growth curves of *P. aeruginosa* Δ4AQ grown in LB or in LB supplemented with 40 µM HHQ, PQS, or HQNO, or with 1 mM IPTG, as indicated.
