# Supplementary material for: Unravelling the Genome-Wide Contributions of Specific 2-Alkyl-4-Quinolones and PqsE to Quorum Sensing in Pseudomonas aeruginosa
Source: PLoS Pathog. 2016 Nov 16;12(11):e1006029. doi: 10.1371/journal.ppat.1006029 (PMC5112799; doi:10.1371/journal.ppat.1006029)
Supplement: S2 Fig — Pyocyanin production (A) and pqsE RNA levels measured by Real Time PCR (B) in P. aeruginosa ∆pqsA ∆pqsE double mutant strains carrying the pME6032 empty vector or pME6032-derivative plasmids for IPTG-inducible expression of wild type pqsE (pME-pqsE), or pqsE mutated variants lacking the first two codons (pME-pqsE∆1–6) or with a nucleotide insertion after the ATG to alter the protein frame (pME-pqsENoFrame). Culture supernatants and total RNAs are from the indicated strains grown to an OD600 of 1.5 in LB supplemented with 1 mM IPTG. For the Real time PCR analysis, data are normalized to the pqsE RNA level measured in parallel in the P. aeruginosa PAO1 wild type. (PDF) [file ppat.1006029.s005.pdf]

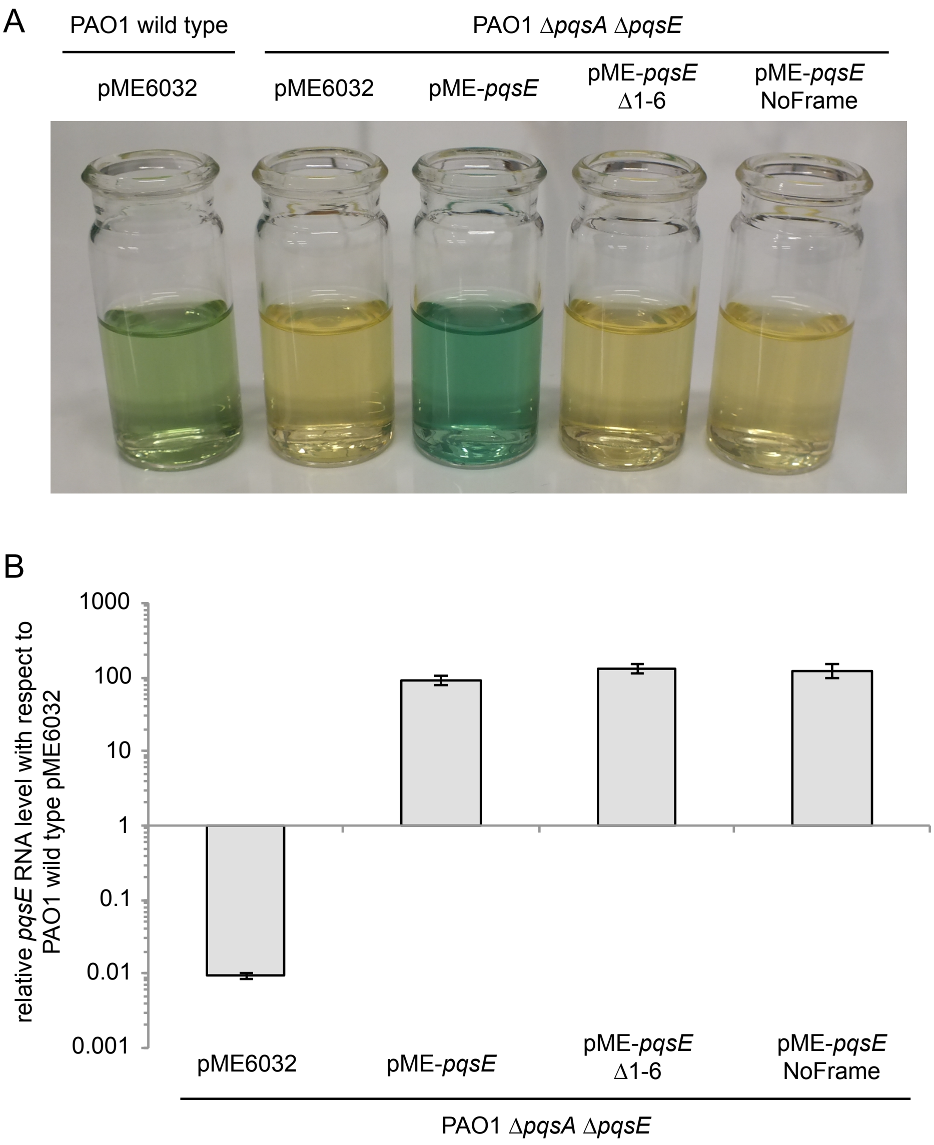

Fig S2. The *pqsE* RNA transcript does not promote pyocyanin production

Pyocyanin production (**A**) and *pqsE* RNA levels measured by Real Time PCR (**B**) in *P. aeruginosa* Δ*pqsA* Δ*pqsE* double mutant strains carrying the pME6032 empty vector or pME6032-derivative plasmids for IPTG-inducible expression of wild type *pqsE* (pME-*pqsE*), or *pqsE* mutated variants lacking the first two codons (pME-*pqsE*Δ1-6) or with a nucleotide insertion after the ATG to alter the protein frame (pME-*pqsE*NoFrame). Culture supernatants and total RNAs are from the indicated strains grown to an OD_{600} of 1.5 in LB supplemented with 1 mM IPTG. For the Real time PCR analysis, data are normalized to the *pqsE* RNA level measured in parallel in the *P. aeruginosa* PAO1 wild type.
