# Supplementary material for: Unravelling the Genome-Wide Contributions of Specific 2-Alkyl-4-Quinolones and PqsE to Quorum Sensing in Pseudomonas aeruginosa
Source: PLoS Pathog. 2016 Nov 16;12(11):e1006029. doi: 10.1371/journal.ppat.1006029 (PMC5112799; doi:10.1371/journal.ppat.1006029)
Supplement: S3 Fig — Amplification of cDNAs retro-transcribed from RNA extracted from (A) P. aeruginosa PAO1 wild type grown in LB and (B) P. aeruginosa ∆4AQ grown in LB supplemented with 1 mM IPTG, to an OD600 of 1.5. A 200 bp DNA region within the pqsE gene (pqsE), a 280 bp DNA region spanning from 97 bp upstream of the pqsE stop codon to 68 bp downstream of the phnA start codon (pqsE-phnA), and a 200 bp DNA region inside the phnA gene (phnA) were amplified from: 1, PAO1 genomic DNA (positive control); 2, cDNA; 3, the corresponding RNA (negative control). L, GeneRuler 100 bp DNA Ladder Plus (MBI Fermentas). (PDF) [file ppat.1006029.s006.pdf]

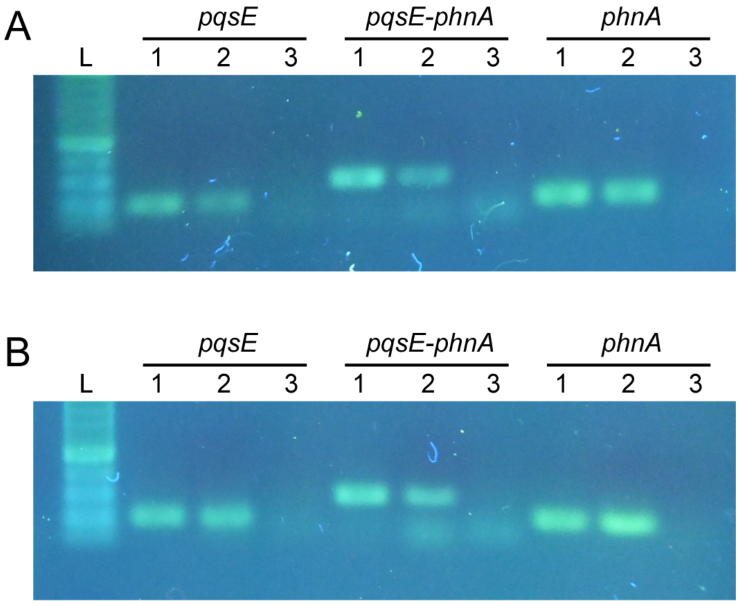

Fig S3. RT-PCR analysis showing co-transcription of *pqsE* and *phnA*

Amplification of cDNAs retro-transcribed from RNA extracted from (**A**) *P. aeruginosa* PAO1 wild type grown in LB and (**B**) *P. aeruginosa* Δ4AQ grown in LB supplemented with 1 mM IPTG, to an OD_{600} of 1.5. A 200 bp DNA region within the *pqsE* gene (*pqsE*), a 280 bp DNA region spanning from 97 bp upstream of the *pqsE* stop codon to 68 bp downstream of the *phnA* start codon (*pqsE-phnA*), and a 200 bp DNA region inside the *phnA* gene (*phnA*) were amplified from: 1, PAO1 genomic DNA (positive control); 2, cDNA; 3, the corresponding RNA (negative control). L, GeneRuler 100 bp DNA Ladder Plus (MBI Fermentas).
