# Supplementary material for: Unravelling the Genome-Wide Contributions of Specific 2-Alkyl-4-Quinolones and PqsE to Quorum Sensing in Pseudomonas aeruginosa
Source: PLoS Pathog. 2016 Nov 16;12(11):e1006029. doi: 10.1371/journal.ppat.1006029 (PMC5112799; doi:10.1371/journal.ppat.1006029)
Supplement: S4 Fig — (A) Anthranilate was quantified by LC-MS/MS analysis in P. aeruginosa wild type and ∆4AQ grown to an OD600 of 1.5 in LB or in LB supplemented with 1 mM IPTG. Standard deviations are based on the mean values of three parallel cultures. (B) Maximal PphzA1 and PphzA2 promoter activity measured in P. aeruginosa PAO1 wild type and in the ∆pqsAHLE strain carrying the pUCP18 empty vector or the pUCPpqsE plasmid for constitutive expression of pqsE. Strains were grown in LB or in LB supplemented with 40 μM HHQ or PQS. Promoter activity is reported as Relative Light Units (RLU)/OD600. (PDF) [file ppat.1006029.s007.pdf]

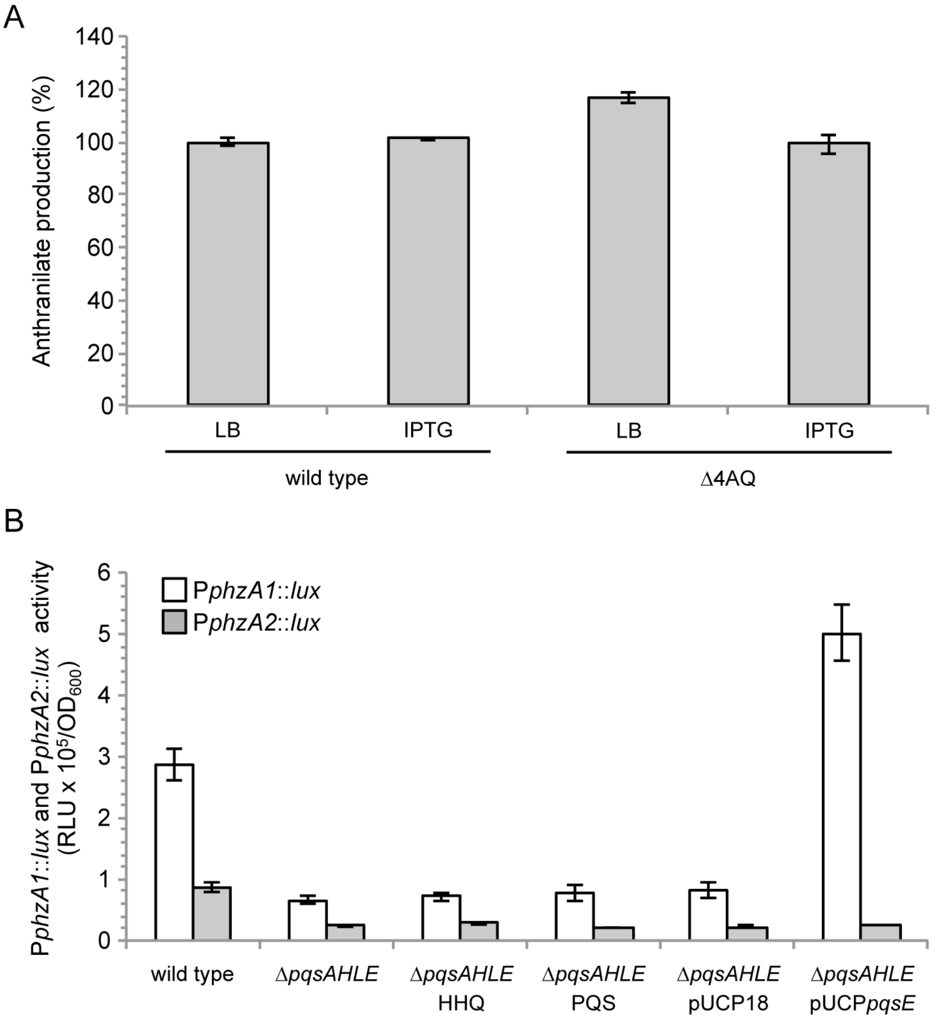

Fig S4. PqsE does not affect anthranilate production and P*phzA2* activity, while it positively controls P*phzA1* activity

(**A**) Anthranilate was quantified by LC-MS/MS analysis in *P. aeruginosa* wild type and Δ4AQ grown to an OD_{600} of 1.5 in LB or in LB supplemented with 1 mM IPTG. Standard deviations are based on the mean values of three parallel cultures. (**B**) Maximal P*phzA1* and P*phzA2* promoter activity measured in *P. aeruginosa* PAO1 wild type and in the Δ*pqsAHLE* strain carrying the pUCP18 empty vector or the pUCP*pqsE* plasmid for constitutive expression of *pqsE*. Strains were grown in LB or in LB supplemented with 40 µM HHQ or PQS. Promoter activity is reported as Relative Light Units (RLU)/OD_{600}.
