# Supplementary material for: Unravelling the Genome-Wide Contributions of Specific 2-Alkyl-4-Quinolones and PqsE to Quorum Sensing in Pseudomonas aeruginosa
Source: PLoS Pathog. 2016 Nov 16;12(11):e1006029. doi: 10.1371/journal.ppat.1006029 (PMC5112799; doi:10.1371/journal.ppat.1006029)
Supplement: S5 Fig — Maximal promoter activity measured in P. aeruginosa ∆4AQ strains carrying the transcriptional fusions (A) PpqsH::lux or (B) PpqsL::lux. Strains were grown in LB or in LB supplemented with 40 μM AQs or 1 mM IPTG, as indicated below the graphs. Promoter activity is reported as Relative Light Units (RLU)/OD600. (PDF) [file ppat.1006029.s008.pdf]

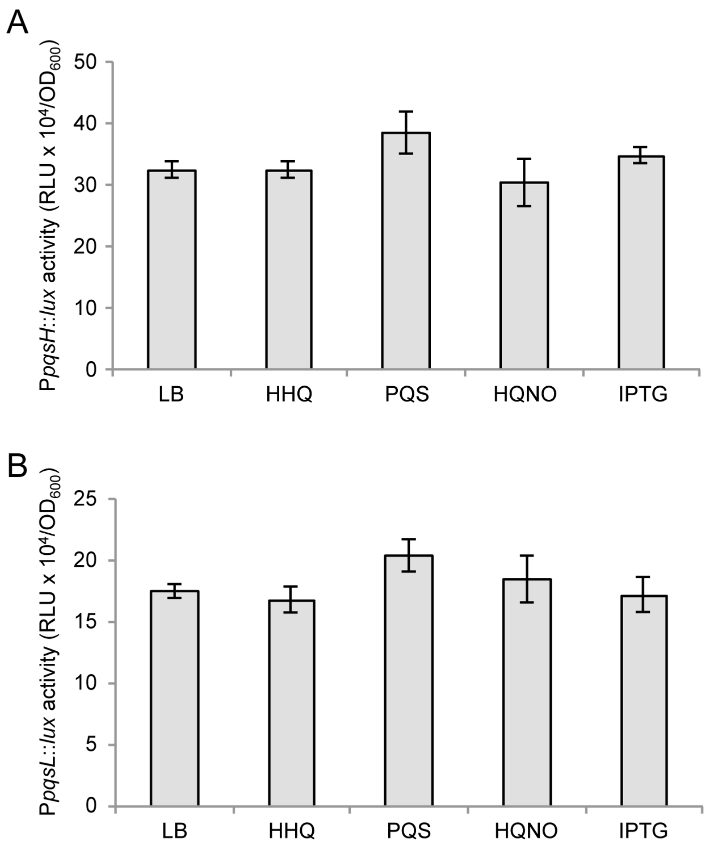

Fig S5. AQs and PqsE do not affect P*pqsH* and P*pqsL* activity

Maximal promoter activity measured in *P. aeruginosa* Δ4AQ strains carrying the transcriptional fusions (**A**) P*pqsH*::*lux* or (**B**) P*pqsL*::*lux*. Strains were grown in LB or in LB supplemented with 40 μM AQs or 1 mM IPTG, as indicated below the graphs. Promoter activity is reported as Relative Light Units (RLU)/OD_{600}.
