# Supplementary material for: Unravelling the Genome-Wide Contributions of Specific 2-Alkyl-4-Quinolones and PqsE to Quorum Sensing in Pseudomonas aeruginosa
Source: PLoS Pathog. 2016 Nov 16;12(11):e1006029. doi: 10.1371/journal.ppat.1006029 (PMC5112799; doi:10.1371/journal.ppat.1006029)
Supplement: S6 Fig — Maximal promoter activity in strains carrying the transcriptional fusions PpqsA::lux (A and B) or PpqsR::lux (C and D). Strains were grown in LB or in LB supplemented with 40 μM PQS, 500 μM 2,2’-dipyridyl (DIP), or 160 μM deferiprone (DEF), as indicated. Diamonds indicate the pyoverdine levels measured in parallel in culture supernatants. Promoter activity is reported as Relative Light Units (RLU)/OD600; pyoverdine levels are reported as OD405 normalized to cell density (OD600). (PDF) [file ppat.1006029.s009.pdf]

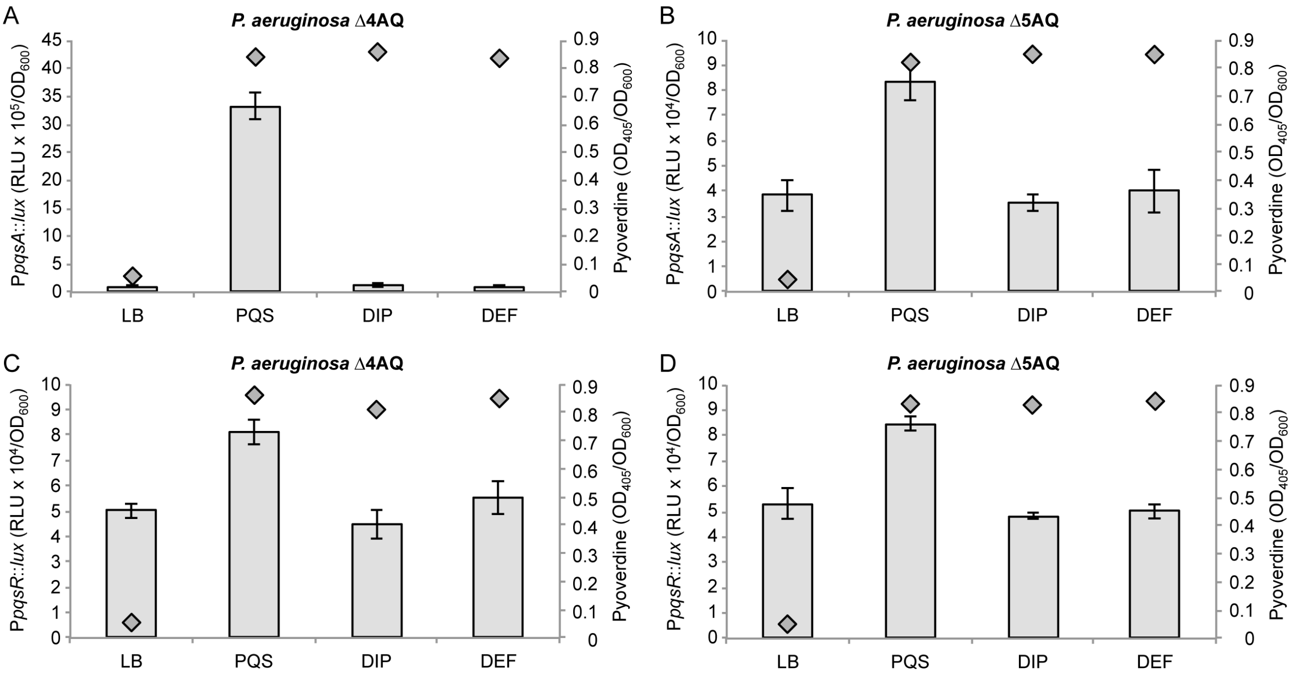

Fig S6. The iron-chelators 2,2'-dipyridyl and deferiprone do not increase P*pqsA* activity

Maximal promoter activity in strains carrying the transcriptional fusions P*pqsA*::*lux* (**A** and **B**) or P*pqsR*::*lux* (**C** and **D**). Strains were grown in LB or in LB supplemented with 40 µM PQS, 500 µM 2,2'-dipyridyl (DIP), or 160 µM deferiprone (DEF), as indicated. Diamonds indicate the pyoverdine levels measured in parallel in culture supernatants. Promoter activity is reported as Relative Light Units (RLU)/OD_{600}; pyoverdine levels are reported as OD_{405} normalized to cell density (OD_{600}).
