# Supplementary material for: Unravelling the Genome-Wide Contributions of Specific 2-Alkyl-4-Quinolones and PqsE to Quorum Sensing in Pseudomonas aeruginosa
Source: PLoS Pathog. 2016 Nov 16;12(11):e1006029. doi: 10.1371/journal.ppat.1006029 (PMC5112799; doi:10.1371/journal.ppat.1006029)
Supplement: S7 Fig — (A-D) Maximal promoter activity in the strains carrying the transcriptional fusions PpqsA::lux (A and B) or PpqsR::lux (C and D). White bars indicate the pvdS-proficient genetic backgrounds (∆4AQ and ∆5AQ); grey bars indicate the pvdS-mutant genetic backgrounds (∆4AQ∆pvdS and ∆5AQ∆pvdS). Strains were grown in LB or in LB supplemented with 40 μM HHQ or PQS, as indicated. Diamonds indicate the pyoverdine levels measured in culture supernatants in the pvdS-proficient (white diamonds) or pvdS-mutant (grey diamonds) genetic backgrounds. Promoter activity is reported as Relative Light Units (RLU)/OD600; pyoverdine levels are reported as OD405 normalized to cell density (OD600). (E) Maximal PpqsA::lux and PpqsR::lux promoter activity in the wild type (white bars) and ∆pvdS (grey bars) strains grown in LB. Promoter activity is reported as Relative Light Units (RLU)/OD600. (F) E. coli H1717 cells containing the plasmids indicated and grown for 24 h at 37°C on McConkey agar supplemented with 10 μg ml-1 Tc and 20 μM FeSO4. Red-staining indicates the ability to ferment lactose and hence the binding of Fur to the target promoter. miniCTX-PpchR::lux, positive control (red colonies); miniCTX-lux, negative control (white colonies). (PDF) [file ppat.1006029.s010.pdf]

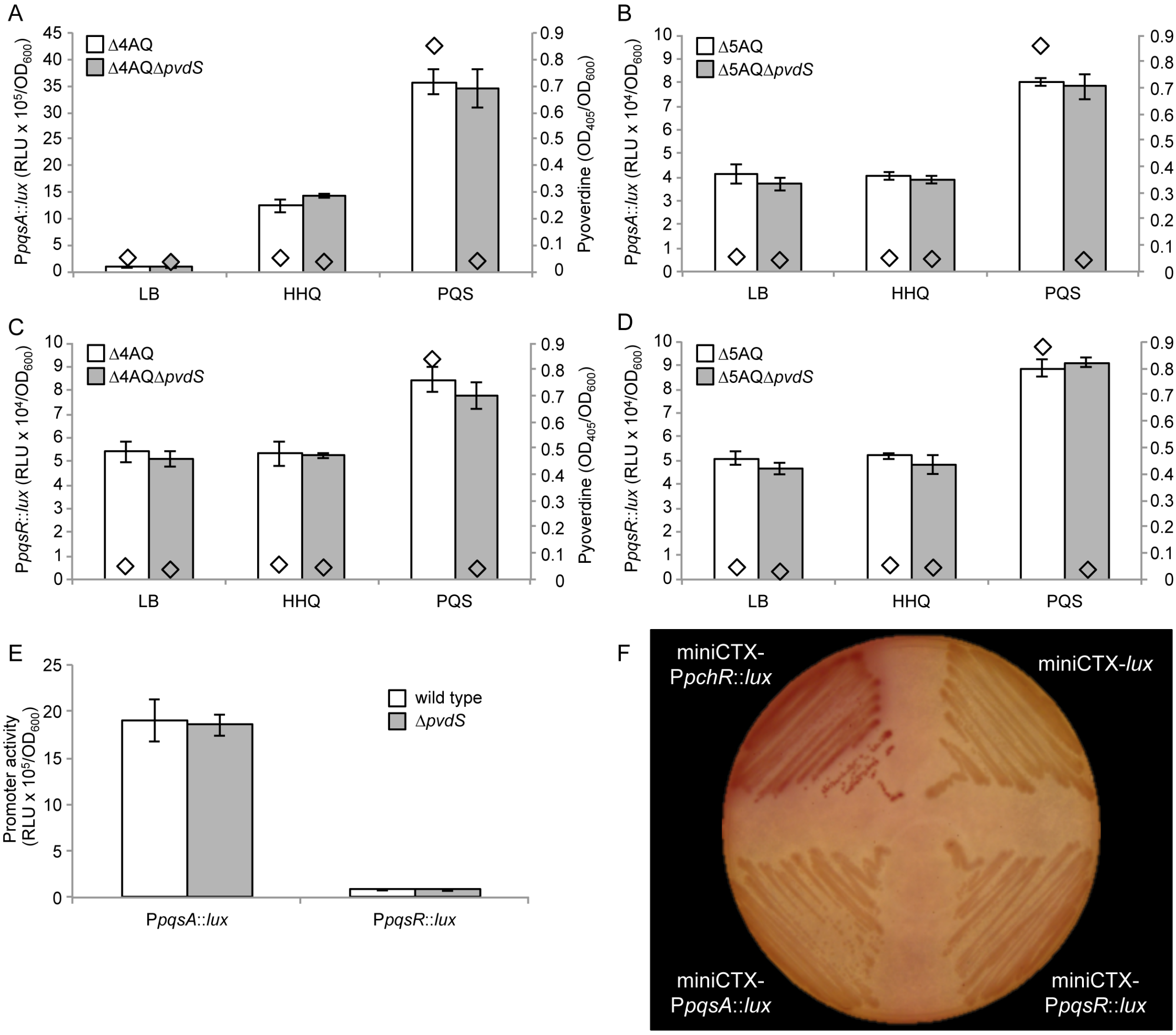

Fig S7. Impact of Fur and PvdS on the P*pqsA* and P*pqsR* promoter regions

(**A**-**D**) Maximal promoter activity in the strains carrying the transcriptional fusions P*pqsA*::*lux* (**A** and **B**) or P*pqsR*::*lux* (**C** and **D**). White bars indicate the *pvdS*-proficient genetic backgrounds (Δ4AQ and Δ5AQ); grey bars indicate the *pvdS*-mutant genetic backgrounds (Δ4AQΔ*pvdS* and Δ5AQΔ*pvdS*). Strains were grown in LB or in LB supplemented with 40 µM HHQ or PQS, as indicated. Diamonds indicate the pyoverdine levels measured in culture supernatants in the *pvdS*-proficient (white diamonds) or *pvdS*-mutant (grey diamonds) genetic backgrounds. Promoter activity is reported as Relative Light Units (RLU)/OD_{600}; pyoverdine levels are reported as OD_{405} normalized to cell density (OD_{600}). (**E**) Maximal P*pqsA*::*lux* and P*pqsR*::*lux* promoter activity in the wild type (white bars) and Δ*pvdS* (grey bars) strains grown in LB. Promoter activity is reported as Relative Light Units (RLU)/OD_{600}. (**F**) *E. coli* H1717 cells containing the plasmids indicated and grown for 24 h at 37ºC on McConkey agar supplemented with 10 µg ml^{-1} Tc and 20 µM $FeSO_4$. Red-staining indicates the ability to ferment lactose and hence the binding of Fur to the target promoter. miniCTX-P*pchR*::*lux*, positive control (red colonies); miniCTX-*lux*, negative control (white colonies).
